# ATH-1105, a small-molecule positive modulator of the neurotrophic HGF system, is neuroprotective, preserves neuromotor function, and extends survival in preclinical models of ALS

**DOI:** 10.3389/fnins.2024.1348157

**Published:** 2024-02-08

**Authors:** Andrée-Anne Berthiaume, Sherif M. Reda, Kayla N. Kleist, Sharay E. Setti, Wei Wu, Jewel L. Johnston, Robert W. Taylor, Liana R. Stein, Hans J. Moebius, Kevin J. Church

**Affiliations:** Athira Pharma, Inc., Bothell, WA, United States

**Keywords:** ATH-1105, ALS, hepatocyte growth factor, neuroprotection, neurotrophic factor, neurofilament light chain, small-molecule therapeutics, TDP-43

## Abstract

**Introduction:**

Amyotrophic lateral sclerosis (ALS), a progressive and fatal neurodegenerative disorder, primarily affects the motor neurons of the brain and spinal cord. Like other neurodegenerative conditions, ongoing pathological processes such as increased inflammation, excitotoxicity, and protein accumulation contribute to neuronal death. Hepatocyte growth factor (HGF) signaling through the MET receptor promotes pro-survival, anti-apoptotic, and anti-inflammatory effects in multiple cell types, including the neurons and support cells of the nervous system. This pleiotropic system is therefore a potential therapeutic target for treatment of neurodegenerative disorders such as ALS. Here, we test the effects of ATH-1105, a small-molecule positive modulator of the HGF signaling system, in preclinical models of ALS.

**Methods:**

*In vitro*, the impact of ATH-1105 on HGF-mediated signaling was assessed via phosphorylation assays for MET, extracellular signal–regulated kinase (ERK), and protein kinase B (AKT). Neuroprotective effects of ATH-1105 were evaluated in rat primary neuron models including spinal motor neurons, motor neuron-astrocyte cocultures, and motor neuron-human muscle cocultures. The anti-inflammatory effects of ATH-1105 were evaluated in microglia- and macrophage-like cell systems exposed to lipopolysaccharide (LPS). *In vivo*, the impact of daily oral treatment with ATH-1105 was evaluated in Prp-TDP43^A315T^ hemizygous transgenic ALS mice.

**Results:**

*In vitro*, ATH-1105 augmented phosphorylation of MET, ERK, and AKT. ATH-1105 attenuated glutamate-mediated excitotoxicity in primary motor neurons and motor neuron- astrocyte cocultures, and had protective effects on motor neurons and neuromuscular junctions in motor neuron-muscle cocultures. ATH-1105 mitigated LPS-induced inflammation in microglia- and macrophage-like cell systems. *In vivo*, ATH-1105 treatment resulted in improved motor and nerve function, sciatic nerve axon and myelin integrity, and survival in ALS mice. Treatment with ATH-1105 also led to reductions in levels of plasma biomarkers of inflammation and neurodegeneration, along with decreased pathological protein accumulation (phospho-TDP-43) in the sciatic nerve. Additionally, both early intervention (treatment initiation at 1 month of age) and delayed intervention (treatment initiation at 2 months of age) with ATH-1105 produced benefits in this preclinical model of ALS.

**Discussion:**

The consistent neuroprotective and anti-inflammatory effects demonstrated by ATH-1105 preclinically provide a compelling rationale for therapeutic interventions that leverage the positive modulation of the HGF pathway as a treatment for ALS.

## Introduction

Amyotrophic lateral sclerosis (ALS) is a progressive and ultimately fatal neurodegenerative disorder characterized by selective motor neuron loss in the brain and spinal cord (Hulisz, 2018). In the United States, the incidence of ALS is 1–2.6 per 100,000 cases annually (Talbott et al., 2016). The prognosis for individuals diagnosed with ALS is poor; symptoms rapidly interfere with quality of life, and the majority of affected individuals succumb to the disease within 5 years after diagnosis (Tortelli et al., 2012; Talbott et al., 2016; Hulisz, 2018). Of those with ALS, approximately 90% have sporadic (idiopathic) ALS and no known family history of the disease, whereas the other 10% have a familial form of ALS driven by inherited genetic mutations (Brown and Al-Chalabi, 2017). Although it is likely that multiple factors contribute to the onset and progression of ALS, one hallmark is the presence of trans-activation response DNA binding protein-43 (TDP-43)–positive extranuclear inclusions in the affected neurons, which is identified in 97% of all ALS cases (Scotter et al., 2015; Jeon et al., 2019). Under normal physiological conditions, TDP-43 is a highly conserved and ubiquitously expressed RNA/DNA-binding protein that has versatile functions, including roles in transcription, translation, mRNA transport and stabilization, miRNA processing, and stress granule formation (Cohen et al., 2011; Prasad et al., 2019). Abnormal cleavage, hyperphosphorylation, ubiquitination, and fragmentation are associated with nuclear depletion and extranuclear localization of the TDP-43 protein, which is a major pathological finding in people with ALS and points to the dysfunction of TDP-43 as a critical component of the disease (Buratti and Baralle, 2008; Kabashi et al., 2008; Buratti, 2015; Palomo et al., 2019; Jo et al., 2020).

In addition to the characteristic presence of extranuclear TDP-43, ALS is associated with multiple pathological processes that ultimately contribute to motor neuron dysfunction and death (Liu and Wang, 2017; van den Bos et al., 2019; Vallarola et al., 2020). Although not fully understood, several pathogenic mechanisms, including heightened excitotoxicity, mitochondrial dysfunction, oxidative stress, activation of apoptotic pathways, and inflammation, are associated with the disease (Obrador et al., 2020). The impact of these pathological processes and the corresponding disease progression can be monitored by assessing several known biomarkers. Levels of proinflammatory cytokines, including interleukin 6 (IL-6) and tumor necrosis factor α (TNF-α), are elevated in the plasma and cerebrospinal fluid (CSF) of people with ALS, reinforcing the role that systemic inflammation may play in the disease mechanism and progression (Tortelli et al., 2020). Neurofilament light chain (NfL), a structural protein found in axons, is released into the CSF and plasma at levels proportional to the degree of ongoing axonal damage, thereby serving as a biomarker of neurodegeneration in neurological conditions, including ALS (Gaetani et al., 2019; Verde et al., 2019). These biomarkers have potential clinical usefulness as diagnostic, prognostic, and pharmacodynamic indicators, and therefore may relay evidence of disease modification in response to pharmacological intervention (Gaetani et al., 2019; Staats et al., 2022). In addition, IL-6, TNF-α, and NfL levels are elevated in preclinical ALS models, and therefore are translationally relevant to helping evaluate the treatment potential of novel therapeutic agents (Yoshihara et al., 2002; Loeffler et al., 2020; Buck et al., 2022; Yang et al., 2022).

Given the wide array of pathological processes associated with ALS, any treatment aiming to effectively address its complex pathophysiology would likely benefit from having multimodal capabilities. The neurotrophic hepatocyte growth factor (HGF) system, signaling through its sole receptor MET, is a potent pro-survival axis that is active during tissue repair and regeneration (Vallarola et al., 2020). The HGF/MET system actuates pleiotropic neuroprotective signaling pathways, leading to potent anti-inflammatory, anti-apoptotic, and pro-survival effects in neurons (Vallarola et al., 2020; Desole et al., 2021). Activation of the neurotrophic HGF system also plays a role in synaptogenesis and neuroplasticity, which may be critical for the recovery of damaged neuronal networks (Eagleson et al., 2016; Hua et al., 2022; Johnston et al., 2023). Literature additionally points to potential involvement of HGF/MET signaling in Schwann cell–mediated nerve repair programs, which provide the myelination of peripheral nerves (Ko et al., 2018; Desole et al., 2021). Based on these multimodal effects, the HGF signaling system as a pharmacological target may be well-positioned to deliver therapeutic benefit to people with ALS. Indeed, previous studies using intrathecal delivery of HGF or HGF overexpression plasmids showed marked delays in ALS disease progression in SOD1^G93A^ mice and rats (Ishigaki et al., 2007; Lee et al., 2019). Lending further validity to this approach, two HGF-based agents are being studied in clinical trials for use in patients with ALS: an HGF plasmid given through a series of intramuscular injections and a recombinant HGF protein injected intrathecally (Sufit et al., 2017; Warita et al., 2019). However, intrathecal and intramuscular drug delivery methods are invasive and confined to one anatomical compartment, which may limit systemic benefit. To address this limitation, we developed a series of small-molecule positive modulators of the neurotrophic HGF signaling system for systemic delivery. One of these, ATH-1105, is in a class of compounds with oral bioavailability and distribution properties favorable for clinical development, including sufficient blood–brain barrier permeability (Taylor et al., 2022). Based on these properties, ATH-1105 is expected to have therapeutic action in all anatomical compartments involved in ALS—the central nervous system (CNS), peripheral nervous system, neuromuscular junctions (NMJs), and muscles. Here, we evaluated the neurotrophic, neuroprotective, and anti-inflammatory impact of ATH-1105 treatment in primary neuron cultures exposed to neurotoxic insults consistent with the pathogenesis of ALS. We then assessed the preclinical efficacy of ATH-1105 in preserving neuromuscular function and extending survival *in vivo* using the Prp-TDP43^A315T^ transgenic mouse model of ALS.

## Methods

### MET, ERK, and AKT phosphorylation assays in HEK293 cell culture

Human embryonic kidney 293 (HEK293; ATCC CRL-1573) cells were used for all phosphorylation assays. Cells were grown at 37° C, 5% CO_2_ in Dulbecco’s Modified Eagle Medium (DMEM) + 10% fetal bovine serum (FBS) until approximately 90% confluent.

To assess the effect of ATH-1105 on HGF-dependent MET activation, HEK293 cells were treated with treatment cocktails consisting of recombinant HGF protein (1 ng/mL; R&D Systems, Minneapolis, MN) with or without ATH-1105 at varying concentrations, and MET phosphorylation was quantified by enzyme-linked immunosorbent assay (ELISA; Phospho-MET [Tyr1234/1235] Sandwich ELISA Kit, CST No. 7227C; Cell Signaling Technology, Danvers, MA). In brief, cells were seeded in polystyrene six-well plates for at least 8 h in serum-free growth media. Treatment cocktails were prepared in Eagle’s Minimum Essential Medium +0.1% FBS, incubated at 37°C in 5% CO_2_ for 1 h, and applied to wells in triplicate for 15 min. Cells were then lysed with 180 μL ice-cold radioimmunoprecipitation assay (RIPA) buffer containing a phosphatase inhibitor (PhosphataseArrest, G-Biosciences, St. Louis, MO) on ice for 15 min. A bicinchoninic acid assay was performed on cell lysates to normalize protein concentration across samples. Phospho-MET ELISA was then performed on the resulting cell lysates per manufacturer instructions. Raw absorbance signals were scaled between subthreshold (1 ng/mL HGF, a concentration that does not induce significant MET activation) and saturating (10 ng/mL HGF) controls such that HGF 1 ng/mL = 1 and HGF 10 ng/mL = 10.

To assess the potential activation of ERK or AKT, we used homogeneous time-resolved fluorescence (HTRF) kits to quantify phosphorylation based on the energy transfer between donor and acceptor phospho-ERK (pERK) or phospho-AKT (pAKT) antibodies. HTRF kit processing for pERK (Phospho-ERK [Thr202/Tyr204] cellular Advanced kit, No. 64AERPET, Cisbio Bioassays, France) and pAKT (Phospho-AKT [Ser473] cellular kit, No. 64AKSPET, Cisbio Bioassays) was carried out per manufacturer instructions. HEK293 cells were seeded in polystyrene 96-well tissue culture plates at 50,000 cells/well in 50 μL serum-free growth media and incubated overnight at 37°C in 5% CO_2_. After 18 h of overnight culture, vehicle and treatment cocktails of various concentrations of ATH-1105 were prepared in serum-free media containing 2 ng/mL HGF, 0.2% FBS, and 0.1% DMSO and incubated at 37°C in 5% CO_2_ for 1 h. Overnight media were aspirated, and 50 μL of appropriate treatments was added to the wells in triplicate and incubated for 20 min at 37°C. Treatments were then removed, and cells were lysed with 50 μL of supplemented lysis buffer (prepared per manufacturer protocol) for 30 min at room temperature while shaking. Sixteen microliters of cell lysate were transferred to a low-volume 384-well plate for processing with pERK- and pAKT-specific antibodies. Antibodies were prepared and added per manufacturer instructions: 2 μL of Eu3+ cryptate working antibody (donor) solution and 2 μL of D2 working antibody (acceptor) solution were added to each well and incubated overnight at room temperature. After excitation with light at a wavelength of 317 nm, fluorescence emission at two different wavelengths (665 and 620 nm) were measured on an Envision plate reader (2104 Envision Excite; PerkinElmer, Waltham, MA), and the HTRF ratio of the acceptor and donor emission signals for each well was calculated as follows: HTRF ratio = (665 nm emission/620 nm emission) × 10,000.

### Cortical neuron protection assays in primary cortical neuron culture

The animal protocol performed by Sai Life Sciences Limited (Hyderabad, India) was approved by the Institutional Animal Ethics Committee (IAEC; Sai Life Sciences Limited) and carried out in accordance with the guidelines of the Committee for the Purpose of Control and Supervision of Experiments on Animals (CPCSEA), Government of India. Every effort was made to minimize the number of animals used and their suffering during experimental procedures, including humane killing. The experiment used brain cells harvested from newborn male rat pups (1–3 days after birth) from pregnant Sprague Dawley rats (dams 18–20 days’ gestation; age 12–14 weeks; weight 275–350 g) procured from Vivo Bio Tech Limited (Hyderabad, India). Delivered rat pups were observed daily for health status until use in the study; only newborn pups in good health were used for the study.

Neuroprotective effects of ATH-1105 against neurotoxic injury were examined in cortical neurons. Brain cortices were dissected from rat pups (1–3 days postnatal period) and chopped in DMEM/10% FBS. Tissues were incubated with DNase/papain/trypsin solution for 30 min at 37°C, followed by incubation in collagenase solution for 1 h. Neurons were triturated in complete neurobasal medium and seeded in wells. Cells were maintained at 37°C in 10% FBS/neurobasal medium, then switched to serum-free neurobasal medium supplemented with 2% B-27. Cultures were grown in serum-free medium containing B-27 for 35–40 days. Cells were treated with 5 μL of ATH-1105, vehicle control (0.5% DMSO), or negative control (only DMEM) for 15 min, then subjected to one of the following: 1-methyl-4-phenylpryidinium (MPP^+^; 500 μM), glutamate (25 μM), lipopolysaccharide (LPS; 1 μM), or hydrogen peroxide (H_2_O_2_; 1 μM) for 24 h at 37°C in 5% CO_2_. After 24 h, 30 μL of CellTiter-Glo (Promega Corporation, Madison, WI) reagent was then added and incubated for 15 min. Ultraluminescence was measured using the EnVision plate reader (2,104 Envision Excite; PerkinElmer) and software (Envision workstation, version 1.14.3049.528).

### Glutamate toxicity, mitochondrial integrity, and caspase-3 activation assays in spinal motor neuron culture

The animal protocol was performed by Neuro-Sys SAS (Gardanne, France) and carried out in accordance with the National Research Council Guide for the Care and Use of Laboratory Animals and followed current European Union regulations (Directive 2010/63/EU; agreement number: B1301310). The experiment used rat spinal cord motor neurons harvested from fetuses from pregnant Sprague Dawley rats of 14 days’ gestation. Fetuses were removed from the uterus and immediately placed in ice-cold L15 Leibovitz medium with a 2% penicillin (10,000 U/mL) and streptomycin (10 mg/mL) solution (PS) and 1% bovine serum albumin. The spinal cord motor neurons were cultured as described by Boussicault et al. (2020).

Spinal cords were treated for 20 min at 37°C with trypsin-ethylenediaminetetraacetic acid (0.05% trypsin and 0.02% EDTA). Dissociation was stopped by addition of DMEM with 4.5 g/L of glucose, containing DNAse I grade II (final concentration 0.5 mg/mL) and 10% fetal calf serum (FCS). Cells were mechanically dissociated by three forced passages through the tip of a 10-mL pipette. Cells were then centrifuged at 515 × *g* for 10 min at 4°C, and the supernatant was discarded. The pellet was resuspended in a defined culture medium consisting of Neurobasal medium with a 2% solution of B-27 supplement, 2 mmol/L of L-glutamine, 2% of PS solution, and 10 ng/mL of brain-derived neurotrophic factor (BDNF). Viable cells were counted and seeded at a density of 20,000 cells/well in 96-well plates pre-coated with poly-L-lysine and cultured at 37°C in 5% CO_2_ in an incubator. The medium was changed every 2 days.

On day 13 of culture, cells were treated with vehicle control (culture medium containing 0.1% DMSO and 0.05 ng/mL HGF), HGF (5 ng/mL), or varying concentrations of ATH-1105 (1 μM, 100 nM, 10 nM, 1 nM, 100 pM) combined with HGF 0.05 ng/mL for 15 min and subjected to glutamate (5 μM). The control group was treated with vehicle control only. After 20 min, treatments were reapplied, and the culture was incubated for 24 h at 37°C in 5% CO_2._ After 24 h, the supernatant was discarded, and cells were fixed in a cold solution of ethanol (95%) and acetic acid (5%) for 5 min at −20°C. After permeabilization with 0.1% saponin, cells were incubated for 2 h with a mouse monoclonal antibody anti–microtubule-associated protein 2 (MAP-2) at a dilution of 1:400 in PBS containing 1% FCS and 0.1% saponin and rabbit polyclonal antibody anti-nuclear TDP-43 at a dilution of 1:100 in PBS containing 1% FCS and 0.1% of saponin. After washing, cells were incubated for 1 h at room temperature with Alexa Fluor 488 goat anti-mouse immunoglobulin G (IgG; Thermo Fisher Scientific, Waltham, MA) at the dilution 1:400 in PBS containing 1% FCS and 0.1% saponin (for MAP-2 antibody) and with Alexa Fluor 568 goat anti-rabbit IgG (Thermo Fisher Scientific, Waltham, MA) at a dilution of 1:400 in PBS containing 1% FCS and 0.1% saponin (for TDP-43 antibody). Nuclei were counterstained with the fluorescent dye Hoechst (Sigma-Aldrich, St. Louis, MO).

A separate culture of motor neurons was used to assess the effect of ATH-1105 on mitochondrial integrity and caspase-3 activation. Cells were treated with vehicle (culture medium containing 0.1% DMSO and 0.05 ng/mL HGF) or varying concentrations of ATH-1105 (1 μM, 100 nM, 10 nM) for 15 min and subjected to glutamate (5 μM). Exogenous HGF (0.05 ng/mL) was added to the medium for all conditions. After 20 min, treatments were reapplied, and the culture was incubated for 24 h at 37°C in 5% CO_2._ After 24 h, the supernatant was discarded, and live cells were incubated with culture medium (first half plate) or MitoTracker Red CMXRos (Cell Signaling Technology; second half plate) at 50 nM in culture medium for 45 min at 37°C. After incubation, the cells (whole plate) were fixed with a cold mixture of 4% paraformaldehyde in PBS (pH 7.3) for 20 min at room temperature. The cells were washed twice in PBS and then permeabilized. Nonspecific sites were blocked with a solution of PBS containing 0.1% of saponin and 1% FCS for 15 min at room temperature. The cultures (whole plate) were then incubated with a mouse monoclonal antibody, anti–MAP-2, at a dilution of 1:400 in PBS containing 1% FCS and 0.1% saponin. After washing, cells were incubated in Alexa Fluor 568 goat anti-mouse IgG at a dilution of 1:800 in PBS containing 1% FCS and 0.1% saponin for 1 h at room temperature. To assess caspase-3 activation, cultures (first half of plate) were incubated with a rabbit polyclonal antibody anti–active caspase-3 at a dilution of 1:100 in PBS containing 1% FCS and 0.1% saponin. This antibody was visualized with Alexa Fluor 568 goat anti-rabbit at a dilution of 1:400 in PBS containing 1% FCS and 0.1% saponin for 1 h at room temperature. Nuclei were counterstained with the fluorescent dye Hoechst (Sigma-Aldrich).

For each condition, 30 pictures (representative of all the well area) per well were automatically taken using ImageXpress (Molecular Devices, San Jose, CA) with 20× magnification, using consistent acquisition parameters. From images, analyses were directly and automatically performed by MetaXpress (Molecular Devices). The following end points were assessed: neuron survival (MAP-2 staining, number of neurons), neurite network (MAP-2 staining, total neurite length in μm), extranuclear TDP-43 in MAP-2–positive neurons (overlapping between MAP-2 and extranuclear TDP-43 in μm^2^), functional mitochondria in MAP-2–positive neurons (overlapping between MAP-2 and MitoTracker, in μm^2^), and analysis of active caspase-3 in MAP-2–positive neurons (overlapping between MAP-2 and activated caspase-3, in μm^2^).

### LPS-induced inflammation in BV2 microglia

BV2 microglial cells were used to assess the anti-inflammation effect of ATH-1105. Cells were grown at 37°C in 5% CO_2_ in high-glucose DMEM with 10% fetal bovine serum (FBS) and 1% penicillin G (100 U/mL) and streptomycin (100 mg/L) until approximately 90% confluence.

To evaluate nitic oxide (NO) changes, BV2 microglial cells were seeded in the inner 60 wells of a 96-well plate at 25,000 cells/well the day before the experiment. On the day of the experiment, cells were starved with serum-free DMEM for 1 h before treatment and then subjected to 2-μg/mL LPS stimulation for 45 min. Afterward, ATH-1105 (100 nM or 1 μM) was added and incubated with LPS for 23 h at 37°C in 5% CO_2_. After 24 h, 50 μL of supernatant was collected from each well for NO production measurement using Griess Reagent system (Promega Corporation). Sulfanilamide solution 50 μL and N-1-napthylethylenediamine dihydrochloride 50 μL were added to supernatant and incubated for 5 min at room temperature. The absorbance was measured at 520 nm in a Biotek Synergy HT plate reader (Winooski, VT). The concentrations of NO were calculated from the standard curve generated from the manufacturer’s protocol.

A Luminex mouse multiplex 14 plex assay (custom; Thermo Fisher Scientific, Waltham, MA) was used to evaluate the gene expression upon LPS stimulation in the presence or absence of ATH-1105 in BV2 microglial cells. The QuantiGene Plex gene expression assay (Luminex, Austin, TX) consisted of magnetic Luminex xMAP beads conjugated with a specific capture probe sequence and a target probe set consisting of capture extenders, a label extender, and blocking extenders. Fourteen gene markers were included in this assay (Table 1). On the first day of the assay, lysate buffer 50 μL (Thermo Fisher Scientific, catalog number QS0102) was added to each well of the plate that was described in this section. The plate was then covered and incubated at 54°C for 30 min to lyse the cells. Afterward, lysate (80 μL) was added to the hybridization plate. Then, an 80-μL diluted lysis mix (1:2 mix lysis buffer and nuclease-free water) was added to two wells as a background control. Finally, 20 μL of the working beads mixture—containing nuclease-free water, the lysis mixture, a blocking reagent, proteinase K, and the capture beads—and the probe set was added to each well of the hybridization plate. The hybridization plate was then sealed and incubated for 20 h at 54°C at 600 rpm on a Labnet VorTemp 56 shaking incubator (Edison, NJ). On the second day of the assay, the hybridization plate was removed from the shaking incubator and transferred onto the magnetic separation plate. The plate was washed three times with wash buffer. Then, 100 μL of pre-amplifier solution was added to each well. The plate was sealed and incubated at 50°C at 600 rpm for 1 h. This process was repeated with the amplifier solution and label probe solutions. Streptavidin R-phycoerythrin (SAPE) working reagent was added to each well and the plate was incubated at room temperature at 600 rpm for 30 min. After SAPE incubation, the plate was washed three times using 130 μL of SAPE wash buffer, and 130 μL of SAPE wash buffer was added to each well for plate reading. The plate was sealed and shaken for 3 min at 800 rpm to suspend beads. The plate was read using Luminex xMAP INTELLIFLEX instrument. Analysis was performed with QuantiGene Plex Data Analysis on the Thermo Fisher Connect website. Gene expression for each analyte was normalized by the geometric mean of four housekeeping genes listed in Table 1.

**Table 1:** Luminex mouse multiplex 14 plex assay inflammation panel.

Gene name	Accession symbol
*COX-2*	NM_011198
*IL-1β*	NM_008361
*IL-10*	NM_010548
*iNOS*	NM_010927
*TBP* (housekeeping gene)	NM_013684
*HPRT* (housekeeping gene)	NM_013556
*TNF-α*	NM_013693
*IL-6*	NM_031168
*LDHA* (housekeeping gene)	NM_010699
*COX-1*	JX945976
*GAPDH* (housekeeping gene)	NM_001001303
*BAX*	NM_007527
*BCL-2*	NM_009741
*NLRP3*	NM_145827

### LPS-induced inflammation in THP-1 differentiated macrophages

THP-1 monocytes were used to investigate the effect of ATH-1105 on LPS-induced cytokine release. Cells were grown at 37°C in 5% CO_2_ in high-glucose Roswell Park Memorial Institute (RPMI) medium with 10% FBS, 1% penicillin G (100 U/mL), and streptomycin (100 mg/L) until approximately 90% confluence. To differentiate THP-1 from monocytes and macrophages, cells were seeded in a 96-well plate at 50,000 cells/well in 100 μL of complete medium. Phorbol myristate acetate was added to each well at the final concentration of 100 ng/mL. The cells were then incubated for 3 days at 37°C in 5% CO_2_ for differentiation.

Before treatment and LPS stimulation, THP-1 macrophages were starved with serum-free RPMI at 37°C in 5% CO_2_ for 3 h. The media were then aspirated, and cells were treated with vehicle (RPMI containing 0.05% DMSO, 0.05% FBS, and 0.05 ng/mL HGF) or ATH-1105 (100 nM or 1 μM) in triplicate at 37°C in 5% CO_2_ for 20 min. After 20 min, treatment was removed and 100 μL of LPS at a final concentration of 50 ng/mL was applied to the culture and incubated at 37°C in 5% CO_2_ for 24 h. Cell culture supernatant was collected to quantify pro-inflammatory cytokine (IL-1β, IL-6, TNF-α) levels using the HTRF kit. For each cytokine, 16 μL of supernatant or standards was added to a 384-well low volume plate. Eu3+ cryptate antibody (donor) and XL antibody (acceptor) were diluted 20-fold with detection buffer and mixed. Then, 4 μL of the mixed antibodies was added to each well and incubated overnight at room temperature with agitation. Fluorescence emissions were measured at two different wavelengths, 665 nm and 620 nm, respectively, on a PerkinElmer Envision plate reader. To calculate the signal value for each well, HTRF ratio was calculated as (signal at 665 nm/signal at 620 nm) × 10^4^. The concentrations of each cytokine were calculated from the standard curve generated from the manufacturer’s protocol.

### Glutamate toxicity assay in motor neuron—astrocyte cocultures

Astrocytes were first cultured in 75-cm^2^ flasks in DMEM (PanBiotech, Germany; ref.: P04-03600, batch: 4070921) containing 10% FCS (Invitrogen, Waltham, MA; ref.: 10270106, batch: 2437712), 2% PS (PanBiotech, ref.: P06-07100, batch: 714121), and 1 mM of sodium pyruvate (PanBiotech, ref.: P04-43100, batch: 4381119). Upon confluency, the cells were shaken overnight at 250 rpm at room temperature to eliminate oligodendrocytes (McCarthy and de Vellis, 1980). Cells were then briefly rinsed with pre-warmed phosphate buffered saline (PBS; PanBiotech; ref.: P04-36500, batch: 8551120), dissociated with trypsin–EDTA (PanBiotech, ref.: P10-023100, batch: 2091385), and centrifuged. After resuspension, the cells were plated at a density of 5,000 cells/well in a 96-well plate and cultured at 37°C in 5% CO_2_.

Rat motor neurons were cultured from fetuses of pregnant Wistar rats at 14 days’ gestation, as previously described (Camu and Henderson, 1994). Fetuses were removed from the uterus and spinal cords were isolated and placed in ice-cold Leibovitz 15 (L15; PanBiotech, ref. P04-27055, batch: 8770122) containing 2% PS, 1% bovine serum albumin (BSA; PanBiotech, ref.: P06-1391100, batch: H210914), and DNase I grade II (0.1 mg/mL; PanBiotech, ref.: P60-37780100, batch: H181015). Cells were then dissociated by several rounds of trituration, centrifuged for 5 min on a BSA cushion, and re-suspended in L15 medium. Viable motor neurons were counted in a Neubauer cytometer using the trypan blue exclusion test. The cells were seeded at a density of 30,000 cells/well on a monolayer of astrocytes in a defined culture medium consisting of Neurobasal (Invitrogen, ref.: 11570556, batch: 2508186) supplemented with 2% B27 (Invitrogen, ref.: 11530536, batch: 2415372), 2% horse serum (Sigma-Aldrich, ref.: H1138, batch: 20A015), L-glutamine (2 mM; PanBiotech, ref.: P04-80100, batch: 7511020), 1% PS, and 0.1% β-mercaptoethanol (Gibco, Thermo Fisher Scientific, ref.: 31350010, batch: 1815203). The coculture was incubated at 37°C in 5% CO_2_. On day 12 of culture, coculture motor neurons and astrocytes were pretreated for 20 min with vehicle control (culture medium containing 0.1% DMSO) or varying concentrations of ATH-1105 (1 μM, 100 nM, 10 nM, 1 nM) for 20 min and then subjected to glutamate at a final concentration of 60 μM for 20 min incubation. Then, the medium was removed, treatments were re-applied, and the culture was incubated for 48 h at 37°C in 5% CO_2_ (Vincent et al., 2004).

After 48 h, cells were fixed in a solution of 4% paraformaldehyde (Alpha Aesar, Thermo Fisher Chemicals, ref. J19943, batch: 211457) for 20 min at room temperature. The cells were then permeabilized, and nonspecific sites were blocked with a solution of PBS containing 0.1% saponin (Sigma-Aldrich, ref.: S7900, batch: BCBL8667V) and 1% FCS for 15 min at room temperature. For neuronal end points, cells were incubated with a mouse anti-Hb9 (Developmental Studies Hybridoma Bank [DSHB], Iowa City, IA; ref: 81.5C10-c, batch: 3/10/22) or a mouse anti–choline acetyl transferase antibody (CHAT, Sigma-Alrich, ref.: AMAB91130, batch: MAB-03512) diluted in PBS containing 0.1% saponin and 1% FCS. Both antibodies were revealed by use of Alexa Fluor 488 goat anti-mouse secondary antibody (Molecular Probe; Invitrogen; ref.: A11001, batch: 2318440) diluted in PBS containing 0.1% saponin and 1% FCS for 1 h at room temperature. For astrocyte end points, cells were incubated with a chicken anti–glial fibrillary acidic protein antibody (GFAP; Abcam, Cambridge, UK; ref.: ab4674; batch: GR3201333-2), and a rabbit anti–excitatory amino acid transporter 2 antibody (EAAT2, Abcam; ref.: ab41621; batch: GR3448942-1) diluted in PBS containing 0.1% saponin and 1% FCS. These antibodies were, respectively, revealed by use of Alexa 633 goat anti-chicken (Molecular probe; ref.: A21449; batch: 2335730) and Alexa Fluor 568 goat anti-rabbit (Molecular probe, ref.: A11011, batch: 2277758) antibodies diluted in PBS containing 0.1% saponin and 1% FCS for 1 h at room temperature. Nuclei were counterstained with the fluorescent marker Hoechst (Sigma-Aldrich; ref.: B1155, Batch: 046M4048V). For each condition, 20 pictures (representative of all the well area) were taken using InCell Analyzer 2,200 (GE Healthcare, Chicago, IL) with 20× magnification. All images were taken under consistent conditions. Analysis was performed automatically using Developer software (GE Healthcare). End points were assessed as follows: number of motor neurons (total number of motor neurons positive for Hb9 staining), length of neurites (length of neurite positive for CHAT staining), number of astrocytes (total number of astrocytes positive for GFAP staining), astrocyte reactivity (ratio GFAP staining intensity to total number of astrocytes), EAAT2 expression (total area positive for EAAT2 staining).

### Glutamate toxicity assays in motor neuron—muscle cocultures

Human muscles were prepared according to a previously described method from portions of healthy patient biopsy (Braun et al., 1996). Muscle cells were established from dissociated cells, plated in gelatin-coated (0.1%) 48-well plate (10,000 cells/well), and grown in a proliferating medium consisting of a mixture of 70% minimum essential medium (MEM; PanBiotech, ref.: P04-08500, batch: 2810620), 30% M199 medium (PanBiotech, ref.: P04-07500, batch: 7800619), 2% PS (PanBiotech, ref.: P06-07100, batch: 9300621), 2 mM L-glutamine (PanBiotech, ref.: P04-80100, batch: 7511020), 10% FCS (Invitrogen, ref.: 10270106, batch: 2437712), 10 ng/mL of insulin (Sigma-Aldrich, ref.: I6634, batch: SLCK8624), 10 pg./mL of epithelial growth factor (EGF; Gibco, ref.: PHG0311, batch: 1543285C), and 5 pg./mL fibroblast growth factor basic (FGFb; PeproTech, Cranbury, NJ, ref.: 100-18B, batch: 091808). The cells were incubated at 37°C in 5% CO_2_, and half the media was refreshed every 2 days.

Rat motor neurons and human muscle cells were prepared as previously described (Braun et al., 1996). Briefly, after 1 week of muscle culture, rat motor neurons were cultured from fetuses of pregnant Wistar rats at 13 days’ gestation. The spinal cords with dorsal root ganglia (DRGs) were removed and placed in ice-cold PBS (PanBiotech; ref.: P04-36500, batch: 8551120). Whole transverse slices of spinal cords with four DRGs attached were placed on the muscle monolayer at one explant per well (in center area). Innervated cultures were maintained in innervating medium consisting of 70% MEM, 30% M199 medium, 2% PS, 2 mM L-glutamine, 10% FCS, and 10 ng/mL insulin. After 24 h, neurites were observed growing out of the spinal cord explants, and after 8 days they had made contact with the myotubes and induced the first contractions. After 3 weeks of coculture, innervated fibers were mature, expressed neuromuscular junctions, and contracted with exposure to motor neuron activity. On day 27 of culture, cocultures of motor neurons and muscles were pretreated for 20 min with vehicle (culture medium containing 0.1% DMSO) or varying concentrations of ATH-1105 (1 μM, 100 nM, 10 nM, 1 nM) for 20 min and then subjected to glutamate at a final concentration of 60 μM for 20 min. The medium was then removed, and treatments were re-applied for 48 h at 37°C in 5% CO_2_ (Vincent et al., 2004).

After 48 h, the cells were fixed in a solution of 4% paraformaldehyde (Alpha Aesar, ref. J19943, batch: 211457) for 20 min at room temperature. Cells were then permeabilized and nonspecific sites were blocked with a solution of PBS containing 0.1% saponin (Sigma-Aldrich; ref.: S7900, batch: BCBL8667V) and 1% FCS for 15 min at room temperature. Cells were incubated with α-bungarotoxin coupled with Alexa 488 (Invitrogen; ref.: B13422; batch: 1986970), a mouse anti-Hb9 (DSHB; ref: 81.5C10-c, batch: 3/10/22), and a rabbit anti-neurofilament (NF; Sigma-Aldrich; ref.: N4142; batch: 0000134031). All antibodies were diluted in PBS containing 1% FCS and 0.1% saponin and incubated for 2 h at room temperature. Hb9 and NF staining were, respectively, revealed with an Alexa Fluor 568 goat anti-mouse (Molecular probe, ref.: A11004, batch: 2332536) and an Alexa Fluor 633 goat anti-rabbit (Molecular probe, ref.: A21070, batch: 2160429) secondary antibody diluted in PBS containing 0.1% saponin and 1% FCS for 1 h at room temperature. Nuclei were counterstained with the fluorescent marker Hoechst (Sigma-Aldrich). For each condition, 20 pictures (representative of all the well area) were taken using InCell Analyzer 2,200 (GE Healthcare) with 20× magnification. All images were taken under consistent conditions. Analysis was performed automatically using Developer software (GE Healthcare). End points were assessed as follows: number of motor neurons (total number of motor neurons positive for Hb9 staining), length of neurites (length of neurites positive for NF staining), number of motor units (number of α-bungarotoxin clusters), acetylcholine receptor clustering (area of α-bungarotoxin clusters).

### Statistical analyses for cell culture assays

For all *in vitro* assays, data for each group were averaged and expressed as mean ± standard error of the mean (SEM). For MET assay, statistical significance was determined by one-way analysis of variance (ANOVA) with Dunnett’s multiple comparison test; comparisons were considered statistically significant when *p* < 0.05 compared with subthreshold HGF (1 ng/mL). For ERK and AKT assays, an unpaired *t* test was used to assess significant differences (*p* < 0.05) between vehicle (2 ng/mL HGF) and ATH-1105 treatment. For the cortical neuron protection assay, statistical significance was determined by one-way ANOVA with Tukey’s test. Comparisons were considered statistically significant when *p* < 0.05 compared with insult only group. For the spinal motor neuron protection assay, statistical significance was determined by one-way ANOVA with Fisher’s least significant differences test. Comparisons were considered statistically significant when *p* < 0.05 compared with glutamate. For inflammation assays, statistical significance was determined by one-way ANOVA with Dunnett’s multiple comparison test. Comparisons were considered statistically significant when *p* < 0.05 versus LPS control. For the coculture studies, statistical significance was determined using a one-way ANOVA followed by Fisher’s least significance differences test. The level of significance was set at *p* < 0.05 versus glutamate control. Post-test significance is reported only for comparisons for which the ANOVA test was significant.

### *In vivo* pharmacokinetic studies in C57BL/6 mice

Pharmacokinetic experiments were performed by Aurigene Pharmaceutical Services Limited (Hyderabad, India) and approved by the Institutional Animal Ethics Committee of Aurigene. To determine the plasma pharmacokinetics after oral delivery, 7- to 10-week-old male C57BL/6 mice received a single oral dose of 10 mg/kg ATH-1105 in 2% DMSO, 20% PEG-400, and 78% saline (vehicle). Blood was collected at 0.16, 0.33, 0.66, 1, 2, 6, 12, and 24 h with potassium EDTA as an anticoagulant. Plasma was separated by centrifugation, and ATH-1105 concentration was determined by liquid chromatography and tandem mass spectrometry (LC/MS/MS; *n* = 3 per time point). To determine brain penetration, a separate group of three male mice were given ATH-1105 5 mg/kg by intravenous (IV) injection. Ten minutes after injection, blood and saline-perfused brain tissue were collected. Drug concentration in plasma and brain was determined by LC/MS/MS. Pharmacokinetic parameters were calculated using WinNonlin 8.0 (Certara, Princeton, NJ).

### *In vivo* assays in the Prp-TDP43^A315T^ mouse model of ALS

#### Animals

ALS *in vivo* studies were conducted using male Prp-TDP43^A315T^ mice and aged-matched wild-type (WT) counterparts. Prp-TDP43^A315T^ mice express mutant TDP-43 cDNA harboring an alanine to threonine substitution at the 315th amino acid (A315T), a substitution associated with familial ALS (Kabashi et al., 2008). Male mice were housed in Makrolon (Covestro AG) cages with filter hoods with continuously filtered air to avoid contamination. Animals were caged in pairs and given *ad libitum* access to water and nutrition (jellified diet required to mitigate gastrointestinal dysfunction in Prp-TDP43^A315T^ mice) (Herdewyn et al., 2014). Cages were kept at a constant temperature with a day/night cycle of 12/12 h. The animal protocol was performed by In Vivex SAS (Lunel, France) and approved by the Animal Studies Committee of Languedoc Roussillon. This protocol complies with French legislation under the European Directives (reference No.: D3417223, APAFIS#23920–2020020320279696 v3). Animal health, disease, and clinical signs were examined daily to ensure that testing procedures were performed only on animals in good health. Experimenters were blind to treatment. For all studies, animals were weighed at study initiation and identified by placing a number on their tails. Before the *in vivo* study phase, randomization was performed using body weight data. Body weight was then measured weekly upon treatment initiation.

The dose optimization study was performed in a single series of 40 male mice, with a total of four treatment groups and 10 mice per group. Each mouse in the group received its respective vehicle or treatment from the age of 1 to 3 months old, for a total of 2 months of treatment. The groups were as follows: WT + vehicle, ALS (Prp-TDP43^A315T^) + vehicle, ALS + ATH-1105 10 mg/kg, and ALS + ATH-1105 20 mg/kg.

The survival study was performed in a single series of 60 male mice, with a total of three treatment groups and 20 mice per group. Each group received their respective vehicle or treatments from the age of 1–5 months old, for a maximum total of 4 months of treatment. All reported mortalities were animals found spontaneously dead in cage. The groups were as follows: WT + vehicle, ALS + vehicle, ALS + ATH-1105 20 mg/kg.

The delayed intervention study was performed in single series of 40 male mice, with a total of four treatment groups, and 10 mice per group. Treatment initiation timing and duration differed amongst the groups in this study. The WT + vehicle group, ALS + vehicle group, and ALS + ATH-1105 20 mg/kg (early) groups received their respective vehicle or treatment from the age of 1 to 4 months old, for a total of 3 months of treatment. The ALS + ATH-1105 20 mg/kg (delayed) group received vehicle from 1 to 2 months old, and then ATH-1105 20 mg/kg from 2 to 4 months old.

#### Motor function tests

For both the dose optimization study and the delayed intervention study, behavioral tests of motor function were performed monthly during the light cycle and consisted of the balance beam, rotarod, grip strength, and Kondziela screen tests in Prp-TDP43^A315T^ mice.

To test their balance and coordination, mice were placed on one end of a narrow beam (3 cm in circumference), which was elevated 50 cm above the ground. For each mouse, the time it took to cross to the other end of the beam was quantified in seconds. Each animal underwent three trials per day at 5-min intervals. Values from the three trials were averaged for each animal and treatment group.

A rotarod apparatus was used to evaluate walking performance, coordination, and balance. Mice were given 1 day of pretraining to become familiarized with the rotarod. On testing days, animals were placed on the rotarod and latency to fall was measured up to a maximum of 300 s, during which the rotation speed was increased from 4 to 40 rpm. Each animal was tested in three trials at each time point, and the values from the trials were averaged for each animal and treatment group.

The neuromuscular strength of the mice was assessed using standardized grip strength tests for all limbs. All-limb grip strength was measured by placing the mouse’s limbs on a horizontal grid connected to a gauge and then pulling the animal’s tail. The maximal force (in newtons) exerted on the grid before the animal’s loss of grip was recorded, and then the mean of three measures was calculated. Data were averaged for each treatment group at each time point.

For the Kondziela screen test, muscular strength and proprioception were assessed. A vertically positioned wire grid box allowed mice to grab onto the grid as they climbed down. The latency to fall (in seconds) was quantified for each mouse. Each animal was tested in three trials per testing day at 5-min intervals. The values from each trial were averaged for each animal and treatment group.

#### Sciatic nerve electrophysiology

For both the dose optimization study and the delayed intervention study, standard electromyography was performed monthly on mice anesthetized with a ketamine-xylazine mixture. A pair of steel-needle electrodes (AD Instruments, Sydney, Australia; MLA1302) was placed subcutaneously along the nerve at the sciatic notch (proximal stimulation). A second pair of electrodes was placed along the tibial nerve above the ankle (distal stimulation). Supramaximal square wave pulses lasting 10 ms at 1 mA were delivered using a PowerLab 26 T (AD Instruments). By use of steel electrodes, compound muscle action potential (CMAP) was recorded from the intrinsic foot muscles. Amplitude and latency of CMAP were determined. The distance between the two sites of stimulation was measured alongside the skin surface with the mice’s legs fully extended, and nerve conduction velocity (NCV) was calculated from latency measurements.

#### Plasma biomarkers

Plasma IL-6, TNF-α, and NfL quantification was determined by ELISA for the dose optimization study and the delayed intervention study. Blood was collected from the submandibular vein into a microtube containing EDTA as an anticoagulant. Samples were centrifuged for 15 min at 1,000 × *g* at 2–8°C within 30 min of collection. Ten microliters of the plasma supernatant were stored at −20°C until analysis. Plasma was diluted at 1:10 in sterile PBS, then IL-6, TNF-α, and NfL quantification by ELISA was performed in duplicate for each animal (Sigma-Aldrich; ref. RAB0308, Sigma-Aldrich ref. RAB0477, and Novus Biologicals, Toronto, Canada, ref. NBP2-80299, respectively).

#### Sciatic nerve immunoassays

For assessment of myelination, the left sciatic nerve of all animals from the dose optimization study was collected and a portion fixed *in situ* for 20 min with 4% paraformaldehyde and 2.5% glutaraldehyde in 0.1 M PBS (pH 7.3). Nerves were then removed and fixed overnight in the same buffer. After washing for 30 min in 0.2 M PBS, the samples were dehydrated using ethanol gradient solutions and were embedded in epoxy resin. Semi-thin cross sections were cut and stained with 0.5% toluidine blue +1% borax +100 mL Milli-Q water (MilliporeSigma, Burlington, MA). The axonal diameter, number of myelinated motor axons per 100 μm^2^, and the myelin g-ratio were quantified using the ImageJ g-ratio plugin (National Institutes of Health and Laboratory for Optical and Computational Instrumentation).

For assessment of phospho-TDP-43 via ELISA, the right sciatic nerve of all animals from the dose optimization study and the delayed intervention study were collected and flash frozen in liquid nitrogen. The frozen tissue was homogenized with sonication in sterile PBS on ice followed by centrifugation (10,000 rpm for 10 min at 4°C). Homogenized samples were tested in the AlphaLISA *SureFire* Human Phospho-TDP-43 ELISA Kit (PerkinElmer, ref. ALSU-PTDP43) following manufacturer’s instructions. Each sample was tested in duplicate at 10 ng/mL total protein, as confirmed by NanoDrop spectrophotometer (Thermo Fisher Scientific). Phospho-TDP-43 was quantified using a fluorescence microplate reader.

For assessment of phospho-TDP-43 via immunohistochemistry in the dose optimization study, following left sciatic nerve fixation, a portion of each sample was incubated for 24–48 h in two successive baths of 6, 20%, then 30% sucrose solution followed by embedment in OCT Embedding Matrix (CellPath, Newton, United Kingdom). Axonal cross-sections (10 μm thick) were cryosectioned, then blocked in blocking buffer (5% fish gelatin and 0.1% triton X-100 in PBS). Axons were marked with chicken anti-mouse Tuj1 (CliniSciences, Nanterre, France, ref. orb94746, 1:200) and detected with goat anti-chicken Alexa Fluor 647 (Thermo Fisher Scientific, ref. A21449, 1:1000). Phosphorylated TDP-43 was marked with rabbit anti-mouse phospho-TDP-43 (Ser409, Ser410) (Invitrogen, ref. PA5-114661, 1:100) and detected with donkey anti-mouse Alexa Fluor 594 (Thermo Fisher Scientific, ref. R37115, 1:1000). All antibodies were diluted in blocking buffer. Images were captured using an LMS700 confocal microscope (Zeiss, Jena, Germany) and phospho-TDP43–aggregated intensities were quantified using the ImageJ plugin.

### Statistical analyses for mouse studies

Statistical significance was determined by one-way ANOVA or two-way ANOVA (or a mixed-effects model if values were missing because of animal death) followed by Dunnett’s multiple comparisons test, which allowed for comparisons between groups under the assumption of normal distribution of the variable and variance homoscedasticity. Statistical significance of the Kaplan–Meier survival probability curves was determined by log-rank (Mantel-Cox) test. Statistical analyses were performed using GraphPad Prism versions 9.1.2 and 10.0.1 for Windows (GraphPad Software, Inc., San Diego, CA). Post-test significance is reported only for comparisons for which the ANOVA test was significant. Comparisons were considered statistically significant when *p* < 0.05 compared with the ALS + vehicle group.

## Results

### ATH-1105 enhanced neurotrophic HGF system signaling through the MET receptor

**Figure 1:**
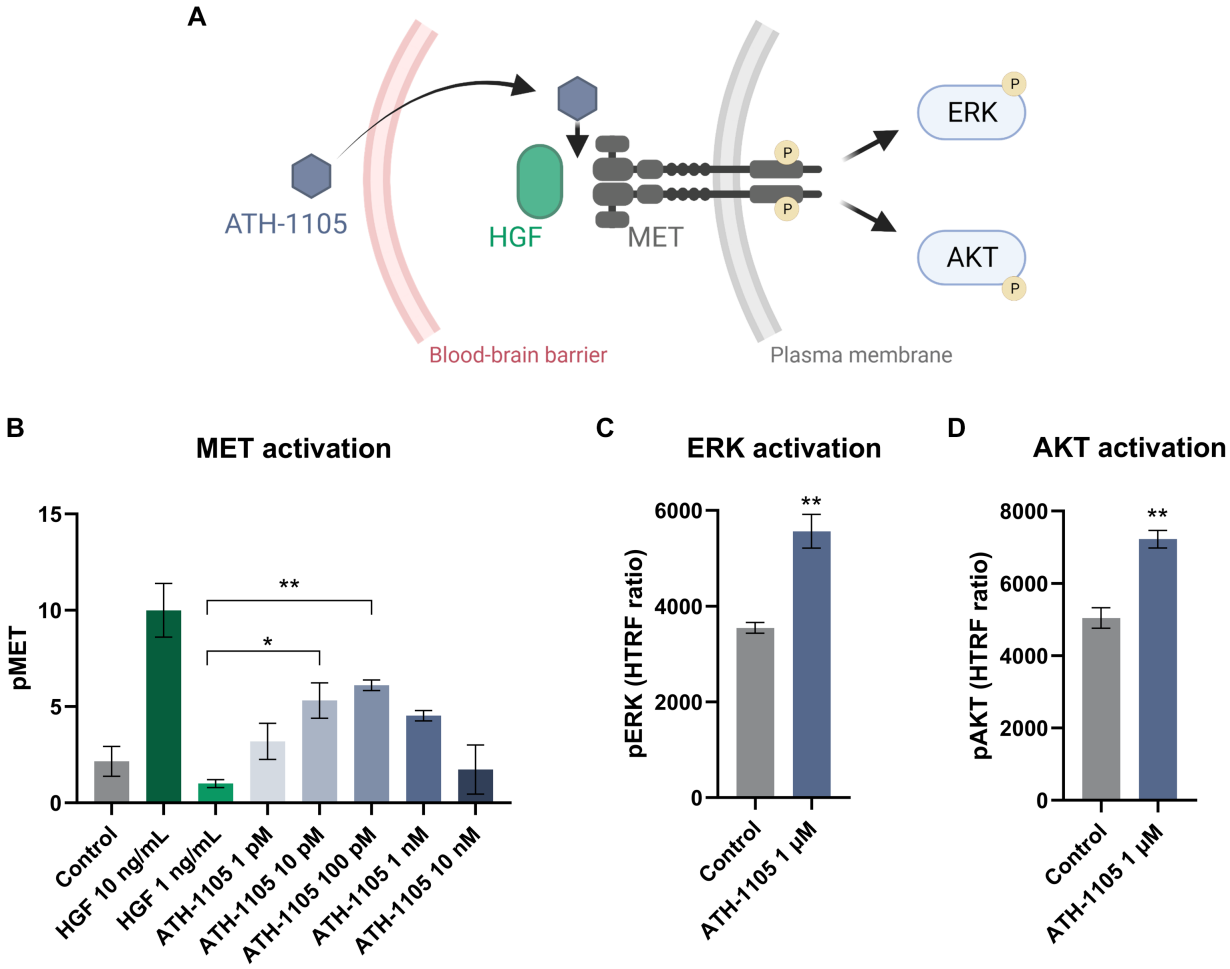
ATH-1105 enhances HGF signaling pathway activation. **(A)** ATH-1105 is a small-molecule positive modulator of the neurotrophic HGF signaling system that crosses the blood–brain barrier and promotes MET phosphorylation (pMET), thereby activating downstream pathways. HEK293 cells treated with HGF + ATH-1105 exhibited a significant increase in the activation of the MET pathway, as measured by phosphorylation of **(B)** MET (pMET), **(C)** ERK (pERK), and **(D)** AKT (pAKT). Data presented as mean ± SEM; *n* = 3 (1 culture) for each assay. For the MET assay, statistical significance was determined by one-way ANOVA with Dunnett’s multiple comparisons; *p < 0.05, **p < 0.01, versus HGF (1 ng/mL). For the ERK and AKT assays, statistical significance was determined by Student’s *t-*test *p < 0.05, **p < 0.01, versus control (HGF 2 ng/mL).

Upon binding of HGF to MET, the MET receptor is activated via transphosphorylation of two catalytic tyrosines (Y1234 and Y1235), subsequently triggering a cascade of intracellular phosphorylation events that initiate downstream signaling pathways (Desole et al., 2021). Therefore, positive modulation of HGF signaling by ATH-1105 is expected to enhance MET phosphorylation and activation of subsequent downstream effectors, such as ERK and AKT (Figure 1A; Desole et al., 2021). To demonstrate that ATH-1105 positively modulates HGF-mediated MET phosphorylation, HEK293 cells were treated with a subthreshold dose of recombinant HGF (1 ng/mL; a dose that does not significantly elevate MET phosphorylation alone) in combination with a range of doses of ATH-1105 (1 pM, 10 pM, 100 pM, 1 nM, 10 nM). HEK293 cells treated with ATH-1105 10 pM and 100 pM + HGF 1 ng/mL exhibited a significant increase in pMET compared with HGF 1 ng/mL alone (Figure 1B). To assess whether positive modulation of HGF signaling by ATH-1105 can mediate HGF-dependent intracellular pathways, we used HTRF to measure pERK and pAKT in HEK293 cells. Treatment with ATH-1105 1 μM led to a significant increase in pERK (Figure 1C) and pAKT (Figure 1D) compared with controls, showing that ATH-1105 can positively modulate HGF-dependent intracellular pathways.

### ATH-1105 was neuroprotective in primary cultures of cortical neurons

It is hypothesized that HGF is neuroprotective against a variety of mechanisms that are central to ALS pathogenesis, such as damage induced by glutamate excitotoxicity and oxidative stress (Desole et al., 2021). To assess whether ATH-1105 could modulate HGF signaling to induce neuroprotective effects, rat cortical neurons were treated with ATH-1105 and subjected to one of the following neurological toxins: glutamate (excitotoxicity), LPS (inflammation), H_2_O_2_ (oxidative stress), or MPP^+^ (mitochondrial dysfunction). Cortical neurons treated with ATH-1105 exhibited significant improvement in cell viability against neuronal damage induced by glutamate (Supplementary Figure S1A), LPS (Supplementary Figure S1B), H_2_O_2_ (Supplementary Figure S1C), and MPP^+^ (Supplementary Figure S1D). The ability of ATH-1105 to enhance survival of cortical neurons across all tested neurotoxic insults suggests that ATH-1105 may protect against multiple cellular and molecular dysfunctions associated with ALS.

### ATH-1105 was neuroprotective in primary cultures of spinal motor neurons

**Figure 2:**
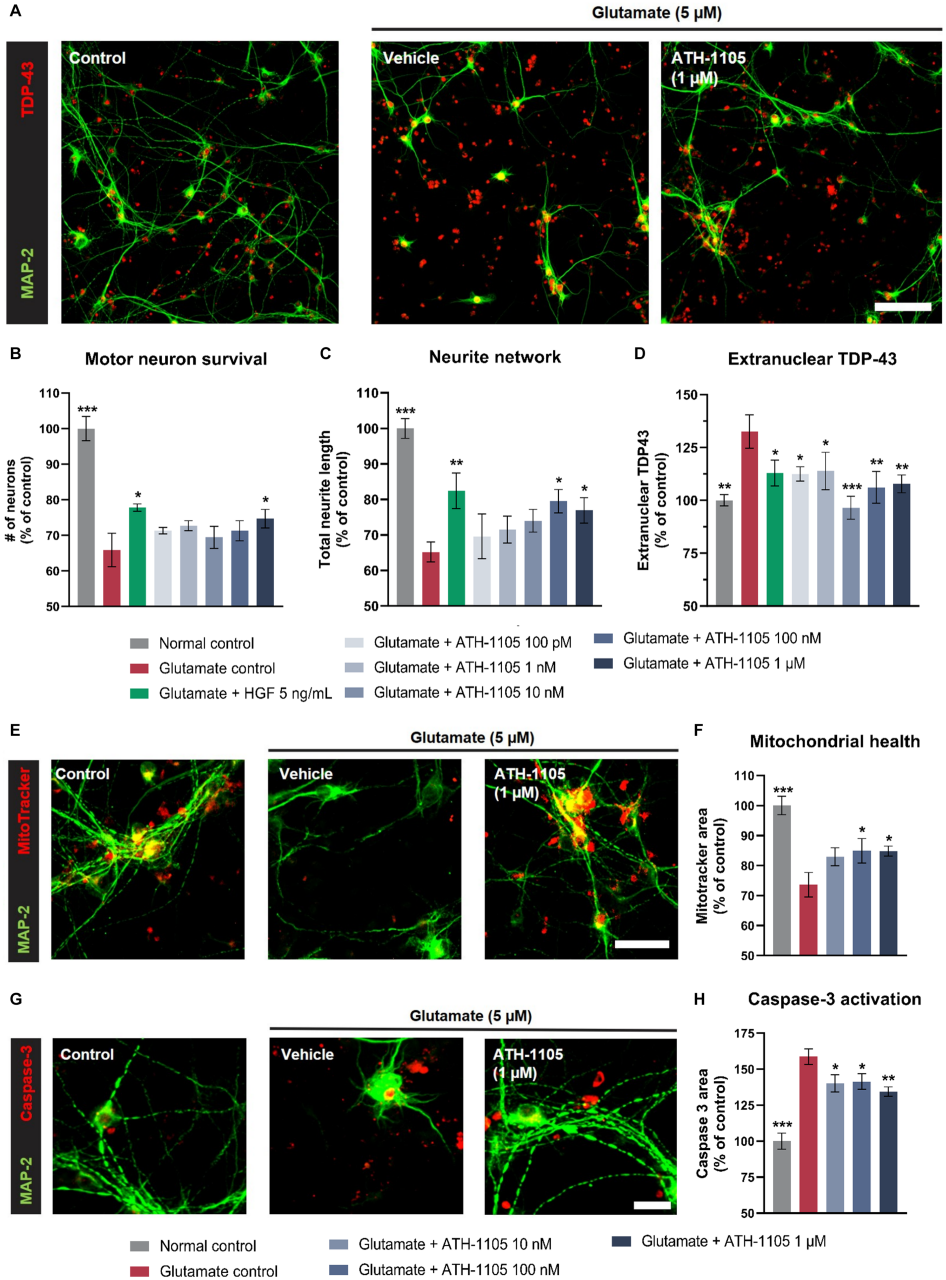
ATH-1105 protects primary cultures of spinal motor neurons against glutamate-mediated toxicity. **(A)** Representative images of spinal motor neurons labeled with MAP-2 and TDP-43 in an immunofluorescence microscopy assay. Scale bar is 100 μm. Quantification of **(B)** neuronal survival, **(C)** neurite length, and **(D)** TDP-43 extranuclear localization in cultures treated with vehicle, HGF, or ATH-1105 in the presence of glutamate (5 μM). **(E)** Representative images of spinal motor neurons labeled with MAP-2 and MitoTracker in an immunofluorescence microscopy assay. Scale bar is 50 μm. Graphical representation of **(F)** functional mitochondria as assessed by MitoTracker after glutamate exposure and treatment with either vehicle or ATH-1105. **(G)** Representative images of spinal motor neurons labeled with MAP-2 and caspase-3 in an immunofluorescence microscopy assay. Scale bar is 25 μm. **(H)** Graphical representation of caspase-3 activation after glutamate exposure and treatment with either vehicle or ATH-1105. All conditions contained 0.05 ng/ml HGF. Data are presented as mean ± SEM; *n* = 5 (1 culture). Statistical significance was determined by one-way ANOVA with Fisher’s LSD. *p < 0.05, **p < 0.01, ***p < 0.001 versus glutamate control.

Although several pathologic signatures contribute to ALS, a consistent hallmark is glutamate-mediated excitotoxicity, which can trigger a cascade of neurodegenerative events that lead to mitochondrial dysfunction, caspase activation, oxidative stress, TDP-43 extranuclear inclusions, neurite degeneration, and motor neuron death (Corona et al., 2007; Boussicault et al., 2020; Obrador et al., 2020). To determine whether ATH-1105 could provide protection from glutamate-induced damage, primary spinal motor neurons were exposed to glutamate in the presence or absence of ATH-1105 and immunoassayed to assess neuronal survival, neurite length, and extranuclear TDP-43 (Figures 2A–D). Spinal motor neurons treated with glutamate + ATH-1105 exhibited significant improvement in neuronal survival (Figure 2B) and preservation of neurite length (Figure 2C) relative to cultures treated with glutamate + vehicle. Furthermore, treatment with ATH-1105 led to a significant decrease in TDP-43 cytoplasmic (extranuclear) localization relative to cultures treated with glutamate + vehicle (Figure 2D), suggesting that positive modulation of HGF by ATH-1105 may promote protective mechanisms that reduce or prevent excitotoxicity-mediated TDP-43 pathology.

To elucidate the mechanisms by which ATH-1105 mediates motor neuron protection, we assessed the effect of ATH-1105 on mitochondrial function and caspase activation in spinal motor neurons subjected to excitotoxic glutamate exposure (Figures 2E–H). Immunofluorescence revealed that spinal motor neurons treated with glutamate + vehicle exhibited a significant decrease in functional mitochondria (Figures 2E,F) and an increase in caspase-3 activation (Figures 2G,H); such effects were significantly attenuated in the presence of ATH-1105. Together, these observations suggest that ATH-1105 confers protection on spinal motor neurons from glutamate-induced damage and that such protection may arise partly from the ability of ATH-1105 to promote mitochondrial health and counteract caspase-3–mediated apoptosis.

### ATH-1105 exhibited anti-inflammatory effects in LPS-activated microglia and macrophages

**Figure 3:**
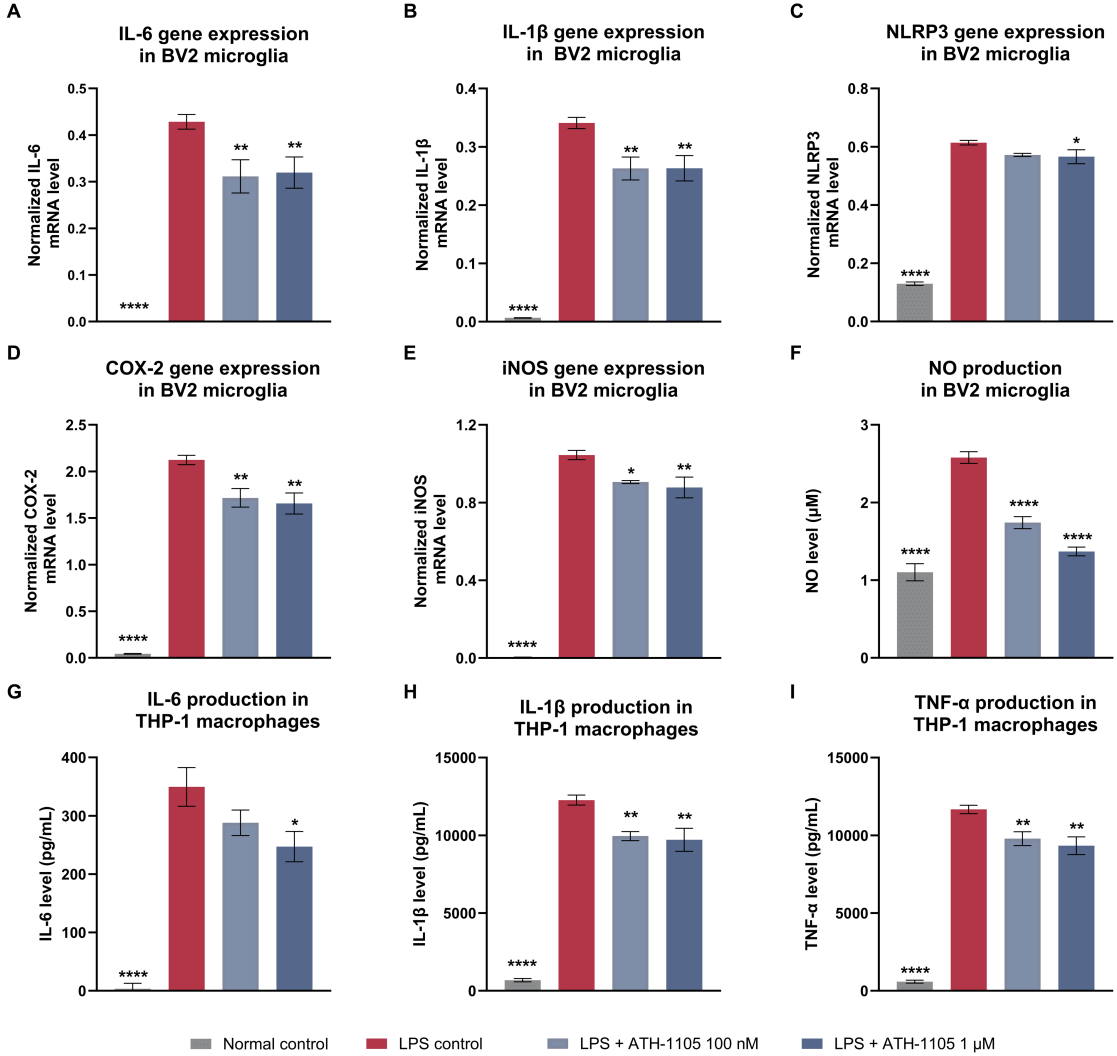
ATH-1105 exerts anti-inflammatory effects in LPS-stimulated BV2 microglia and THP-1–differentiated macrophages. Bar graphs show the normalized gene expression levels of LPS-induced **(A)** IL-6, **(B)** IL-1β, **(C)** NLRP3, **(D)** COX-2, **(E)** iNOS, and **(F)** NO production in BV2 microglial cells in the presence or absence of ATH-1105, and LPS-induced release of **(G)** IL-6, **(H)** IL-1β, and **(I)** TNF-α in THP-1–differentiated macrophages in the presence or absence of ATH-1105. Data are presented as mean ± SEM; *n* = 3 (1 culture). Statistical analysis consisted of one-way ANOVA with Dunnett’s multiple comparisons. *p < 0.05, **p < 0.01, ***p < 0.001, ****p < 0.0001 versus LPS control.

Ongoing inflammation plays a key role in the pathogenesis of ALS, characterized by microglial activation, macrophage infiltration, and excessive production of pro-inflammatory cytokines (Liu and Wang, 2017). Therefore, we investigated the effect of ATH-1105 on LPS-mediated proinflammatory cytokine levels *in vitro* in BV2 microglia cultures (Figures 3A–F). BV2 microglial cells were pre-exposed to LPS and treated with vehicle or ATH-1105 (100 nM or 1 μM) for 23.5 h. Quantification of mRNA of proinflammatory mediators revealed that ATH-1105 significantly reduced LPS-stimulated elevations in gene expression of IL-6 (Figure 3A), IL-1β (Figure 3B), NOD-like receptor protein-3 (NLRP3; Figure 3C), cyclooxygenase-2 (COX-2; Figure 3D), and inducible nitric oxide synthase (iNOS) (Figure 3E). ATH-1105 treatment also reduced NO production compared with the LPS-challenged vehicle control group (Figure 3F).

In addition to neuroinflammation in the CNS, accumulating evidence shows increased activation and invasion of peripheral monocytes into the spinal cord of patients with ALS, resulting in motor neuron damage (Liu et al., 2020). To assess whether ATH-1105 confers anti-inflammatory effects on peripheral macrophages, THP-1 monocytes were differentiated into macrophages and subjected to LPS exposure with or without ATH-1105 and assayed for levels of IL-6, IL-1β, and TNF-α secretion from culture supernatant (Figures 3G–I). ATH-1105 significantly reduced release of IL-6 (Figure 3G), IL-1β (Figure 3H) and TNF-α (Figure 3I) compared with the LPS-challenged vehicle-treated group. The anti-inflammatory effects in microglia and in macrophages *in vitro* highlight the potential for ATH-1105 to mitigate components of central and peripheral inflammation.

### ATH-1105 was neuroprotective in primary coculture models of ALS

**Figure 4:**
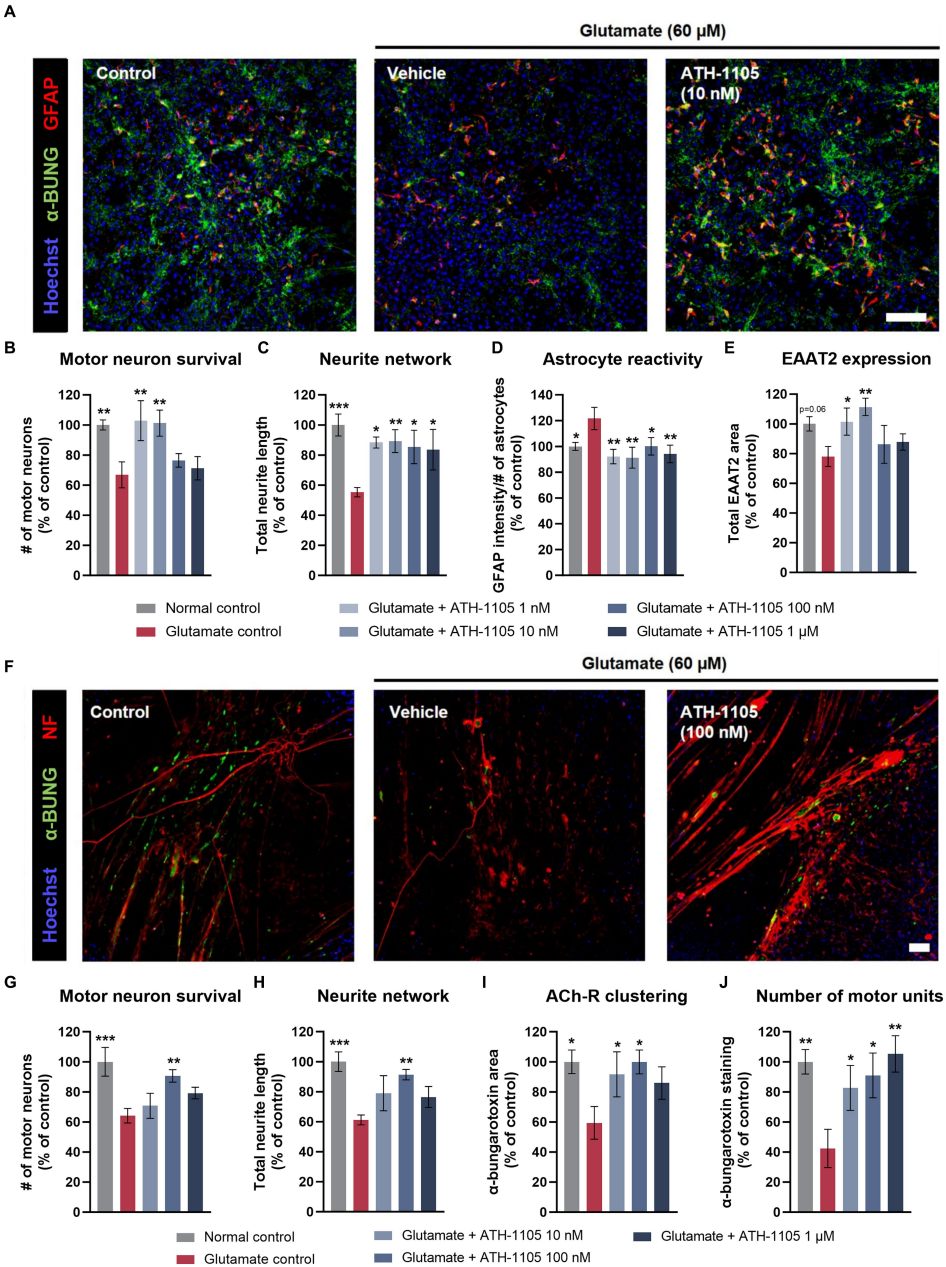
ATH-1105 protects primary motor neuron-astrocyte and motor neuron-muscle cocultures against glutamate-mediated toxicity. **(A)** Representative images of motor neuron–astrocyte coculture labeled with anti-GFAP and anti-EAAT2 in an immunofluorescence microscopy assay. Scale bar is 100 μm. Quantification of **(B)** motor neuron survival, **(C)** neurite network (total length of neurites), **(D)** astrocyte reactivity, and **(E)** EAAT2 expression in cocultures treated with vehicle or ATH-1105, in the presence of glutamate (60 μM). **(F)** Representative images of motor neuron–muscle coculture labeled with anti–α-bungarotoxin (α-bung; as a marker of nicotinic acetylcholine receptors) and anti–neurofilament-200 (as a marker of neuronal processes) in an immunofluorescence microscopy assay. Scale bar is 100 μm. Quantification of **(G)** motor neuron survival, **(H)** neurite network (total length of neurites), **(I)** acetylcholine receptor clustering (α-bung area), and **(J)** number of motor units (number of α-bung staining clusters) in cocultures treated with vehicle or ATH-1105, in the presence of glutamate (60 μM). Data are presented as mean ± SEM; *n* = 6 (1 culture) for each assay. Statistical significance was determined by one-way ANOVA with Fisher’s LSD *p < 0.05, **p < 0.01, ***p < 0.001 versus glutamate control.

Astrocyte dysfunction has been recognized as a key pathological component in the onset and progression of ALS, with astrocyte reactivity and subsequent release of inflammatory mediators contributing substantially to motor neuron degeneration (Vaz et al., 2021). Additionally, dysfunction of EAAT2, which plays a critical role in glutamate reuptake, is well established in ALS and renders neurons highly susceptible to excitotoxic stress (Rosenblum and Trotti, 2017). To assess whether ATH-1105 can address astrocyte dysfunction, primary cocultures of rat motor neurons and astrocytes were treated with glutamate in the presence or absence of ATH-1105 and immunoassayed to assess motor neuron survival, neurite length, astrocyte reactivity, and expression of EAAT2 (Figures 4A–E). Application of glutamate induced a significant loss of motor neurons and neurite length. In contrast, cocultures pretreated with ATH-1105 and exposed to glutamate exhibited a significant rescue of neuronal survival (Figure 4B) and neurite length (Figure 4C) relative to cultures treated with glutamate + vehicle. Additionally, cocultures treated with glutamate + ATH-1105 had significantly decreased astrocyte reactivity (Figure 4D) and increased expression of EAAT2 (Figure 4E) relative to the glutamate + vehicle group. Together, these observations suggest that ATH-1105 may address pathological alterations related to astrocyte dysfunction in ALS.

One of the most prominent pathological hallmarks of ALS is the breakdown and dysfunction of the NMJ (Verma et al., 2022). We used an *in vitro* NMJ model consisting of rat spinal motor neurons in coculture with human muscle to assess the impact of ATH-1105 on NMJ integrity. Cocultures of motor neurons and muscle tissue were treated with glutamate in the presence or absence of ATH-1105 and immunoassayed to assess motor neuron survival, neurite length, acetylcholine receptor clustering (the main neurotransmission system at the NMJ), and number of motor units. Application of glutamate induced a significant loss of motor neurons and neurite length, as well as a significant decrease in acetylcholine receptor clustering and the number of motor units (Figures 4F–J). Pretreatment with ATH-1105, notably at the 100 nM concentration, resulted in a significant increase in neuronal survival (Figure 4G) and preservation of neurite length (Figure 4H), as well as increased acetylcholine receptor clustering (Figure 4I) and number of motor units (Figure 4J) relative to glutamate control cultures. These observations suggest that ATH-1105 may protect NMJs from excitotoxic injury.

### Pharmacokinetic properties of ATH-1105

To enable evaluation of ATH-1105–induced treatment effects *in vivo*, the pharmacokinetic profile of ATH-1105 was determined in male mice. A single oral dose of 10 mg/kg resulted in a maximum plasma concentration (C_max_) of 652 ng/mL and an area under the concentration-time curve from time 0 to infinity (AUC_0-inf_) of 611 ng·h/mL. The compound distributed to the plasma rapidly with a time to maximal concentration (T_max_) of 0.17 h and a half-life of 0.78 h. In a separate study, male mice were given 5 mg/kg ATH-1105 by intravenous injection. After 10 min, blood and perfused brain tissue were collected to determine the brain penetrance. The plasma concentration of ATH-1105 was 1,401 ng/mL and brain concentration was 599 ng/g resulting in a brain to plasma concentration ratio (Kp_brain_) of 0.43. Based on the *in vitro* activity experiments presented (Figures 2–4), the predicted target exposure in the brain is near 100 nM, which is approximately equivalent to an oral dose of ATH-1105 2 mg/kg. A preliminary *in vivo* dose-finding study in a mouse model of ALS was conducted to evaluate the activity of ATH-1105 at 0.4, 2, and 10 mg/kg, which showed moderate activity at 2 mg/kg and superior performance at 10 mg/kg (data not shown). Based on these initial findings, we chose to explore the higher dose range of 10 mg/kg and 20 mg/kg in the following dose optimization study.

### ATH-1105 significantly protected against reduced body weight in a mouse model of ALS

**Figure 5:**
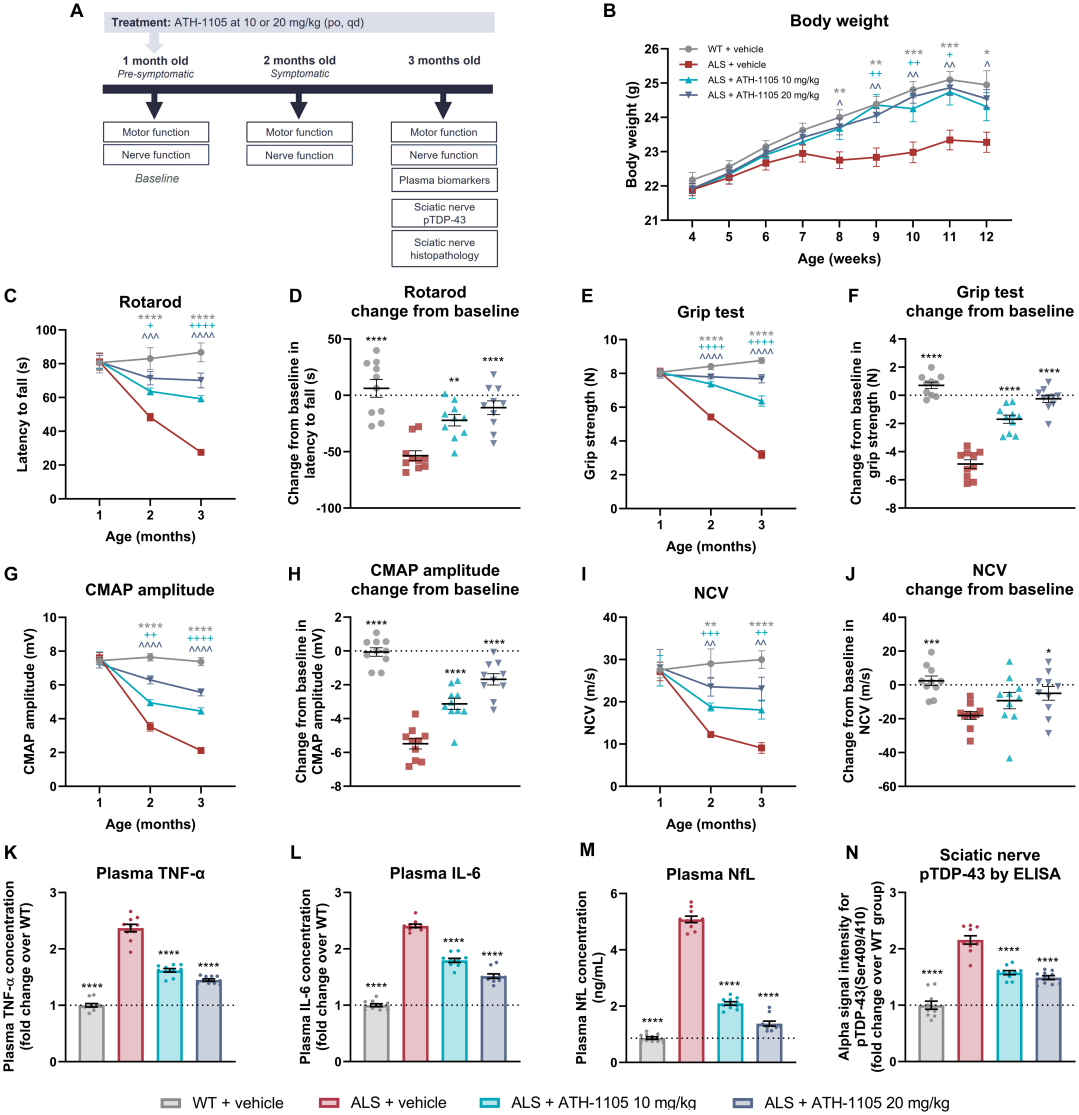
ATH-1105 treatment improves neuromotor function and disease-related biomarkers in ALS mice. **(A)** Experiment timeline. **(B)** Graphical representation of body weight in grams measured every 3 days. Quantification of motor function as measured by **(C)** rotarod over time and as **(D)** change from baseline in latency to fall and as measured by **(E)** grip test over time and as **(F)** change from baseline in grip strength. Quantification of nerve function as indicated by **(G)** CMAP amplitude over time and **(H)** change from baseline in CMAP amplitude, and by **(I)** NCV over time and **(J)** change from baseline in NCV. Plasma levels at 3 months of age of **(K)** TNF-α and **(L)** IL-6 expressed as fold change over the WT group and **(M)** NfL in ng/mL. **(N)** Alpha ELISA signal intensity for pTDP-43 in sciatic nerve homogenates, expressed as fold change over the WT group. Data are presented as mean ± SEM. Statistical significance was determined via **(B,C,E,G,I)** two-way ANOVA or **(D,F,H,J-N)** one-way ANOVA with Dunnett’s multiple comparisons versus ALS + vehicle. “*” Represents WT + vehicle versus ALS + vehicle comparisons, “+” represents ALS + ATH-1105 10 mg/kg versus ALS + vehicle comparisons, and “^” represents ALS + ATH-1105 20 mg/kg versus ALS + vehicle comparisons. The following applies to all symbols: *p < 0.05, **p < 0.01, ***p < 0.001, ****p < 0.0001; *n* = 10 mice per group.

A dose optimization study was designed to determine the effects of daily oral treatment with ATH-1105 for 2 months on ALS-related disease progression in male transgenic Prp-TDP43^A315T^ mice (“ALS mice”) starting at 1 month (4 weeks) of age (Figure 5A). This model was selected based on the previously described relevance of TDP-43 protein pathology to clinical ALS and the progressive development of key ALS-like behavioral and cellular phenotypes observed in these mice (Bargsted et al., 2017). No mortalities were observed over the course of the study.

Although all groups started at comparable body weights, the mice in the ALS + vehicle group began to display persistently lower average body weight than the WT control group beginning at 8 weeks of age. Compared with the ALS + vehicle group, average body weight in ALS mice treated with ATH-1105 was significantly higher from 9 to 11 weeks of age at the 10-mg/kg dose level and from 8 to 12 weeks of age at the 20-mg/kg dose level (Figure 5B). This suggests maintenance of healthy body weight over time in transgenic ALS mice treated with ATH-1105.

### ATH-1105 mitigated motor function deficits in a dose-dependent manner

To determine whether daily treatment with ATH-1105 impacts motor function in ALS mice, motor performance of the mice was evaluated at 1 month of age (“baseline”), 2 months of age (after 1 month of treatment), and 3 months of age (after 2 months of treatment). Four behavioral assessments were conducted at each time point: rotarod latency to fall (Figures 5C,D), grip strength (Figures 5E,F), Kondziela inverted screen latency to fall (Supplementary Figures S2A,B), and balance beam cross time (Supplementary Figures S2C,D). In all metrics, no difference in motor performance across groups was observed at baseline, suggesting that the time point of 1 month of age precedes the development of overt ALS-like motor deficits in male Prp-TDP43^A315T^ mice. The motor function of vehicle-treated ALS mice significantly deteriorated by the next evaluated time point of 2 months of age and continued to worsen through the final time point of 3 months of age. Treatment with ATH-1105 10 mg/kg or 20 mg/kg significantly slowed this functional decline in a dose-dependent manner compared with vehicle treatment in ALS mice at 2 and at 3 months of age in the rotarod (Figure 5C), grip test (Figure 5E), Kondziela screen test (Supplementary Figure S2A), and balance beam test (Supplementary Figure S2C).

To evaluate the magnitude of disease progression over the entire experimental period, change from baseline in motor performance at 3 months of age was calculated. This revealed a significant dose-dependent protection against the development of ALS-related motor dysfunction with ATH-1105 treatment, as measured by rotarod (Figure 5D), grip test (Figure 5F), Kondziela screen test (Supplementary Figure S2B), and balance beam test (Supplementary Figure S2D). For example, in the rotarod test of balance and coordination, for which a shorter latency to fall indicates greater motor impairment, an average change from baseline of −53.58 ± 4.38 s was observed in the ALS + vehicle group over the experimental period, whereas an average loss of only −10.98 ± 6.04 s over time was seen in the ALS mice group treated with ATH-1105 at 20 mg/kg. Similarly, in the grip test assessment for muscular strength, mice in the ALS + vehicle group lost −4.878 ± 0.304 N over time, whereas the ALS mice treated with ATH-1105 at 20 mg/kg only lost an average of −0.25 ± 0.25 N over the experimental time course. These results point to a protection against motor function deterioration related to ALS-like disease processes in Prp-TDP43^A315T^ mice treated with ATH-1105.

### ATH-1105 rescued dysfunctional signal transduction in the sciatic nerve

Electrophysiological assessment of CMAP and NCV is an established, quantitative method in neurological practice to assess the status of peripheral nerve damage and monitor functional decline (Ramroop and Cruz, 2022). To evaluate the impact of ATH-1105 treatment on nerve function, we measured CMAP amplitude and NCV at 1 month, 2 months, and 3 months of age in Prp-TDP43^A315T^ ALS mice (Figures 5G–J). Similar to our observations in the motor function tests, no detectable differences in nerve electrophysiological measurements were found between WT and ALS mice at 1 month of age, thereby allowing us to consider this time point as “baseline.” CMAP amplitude, a measure related to the functionality and integrity of motor units, decreased significantly and progressively in ALS mice treated with vehicle from 2 months of age and onward. Treatment with ATH-1105 10 mg/kg or 20 mg/kg resulted in a significant dose-dependent improvement in the CMAP amplitude deficit at 2 and 3 months of age (Figure 5G). Assessment of change from baseline in CMAP amplitude revealed that the −5.49 ± 0.30 mV reduction in CMAP over time in ALS mice treated with vehicle was reduced to a decrease of only −1.68 ± 0.33 mV in ALS mice treated with ATH-1105 20 mg/kg over the 2-month experimental time course (Figure 5H). This suggests a significant slowing of disease progression in terms of ALS-related neuromuscular dysfunction by ATH-1105. NCV, a measure related to myelination of nerves, also worsened over time in ALS mice, ultimately leading to slower NCV at 2 and 3 months of age and a change from baseline of −18.08 ± 2.32 m/s over time. Treatment with ATH-1105 10 mg/kg or 20 mg/kg led to significantly improved NCV at 2 and 3 months of age (Figure 5I), with a change from baseline of only −4.96 ± 4.07 m/s at the best dose of 20 mg/kg (Figure 5J). This suggests an overall protection of nerve myelination by ATH-1105 in ALS mice, as measured by this functional readout. Together, these electrophysiological measurements indicate preservation of nerve signal transduction in ALS mice treated with ATH-1105.

### ATH-1105 treatment reduced markers of inflammation, neurodegeneration, and protein pathology

To quantify biomarkers related to inflammation and neurodegeneration, plasma was collected at the final experimental time point of 3 months of age after 2 months of daily treatment with vehicle or ATH-1105 10 mg/kg or 20 mg/kg (Figures 5K–M). The plasma concentrations of the pro-inflammatory cytokines significantly increased in the ALS + vehicle group compared with the WT + vehicle group (2.37 ± 0.21-fold increase in TNF-α, and 2.41 ± 0.10-fold increase in IL-6), indicating comparatively high levels of systemic inflammation. Daily treatment with ATH-1105 10 mg/kg or 20 mg/kg for 2 months elicited a significant and dose-dependent decrease in the concentrations of both TNF-α (1.45 ± 0.07-fold change from WT for 20 mg/kg) and IL-6 (1.52 ± 0.13-fold change from WT for 20 mg/kg) in plasma (Figures 5K,L), suggesting reduced inflammation related to ALS-like pathology. Plasma levels of NfL were quantified as an assessment of ongoing neurodegeneration (Figure 5M). As expected, plasma NfL levels were significantly elevated in ALS mice treated with vehicle. While in the WT + vehicle group the average plasma NfL concentration was only 0.87 ± 0.05 ng/mL at 3 months of age, the average level in ALS mice treated with vehicle was 5.08 ± 0.06 ng/mL. In contrast, ALS mice treated with ATH-1105 10 mg/kg or 20 mg/kg had a significant reduction in plasma NfL levels, with average plasma concentrations of 1.38 ± 0.09 ng/mL at the best dose of 20 mg/kg at this same time point. This may indicate decreased neurodegeneration with ATH-1105 treatment in ALS mice.

Last, phosphorylation of TDP-43 (pTDP-43; Ser409/410) in sciatic nerve homogenates was quantified as the driver of ALS-like disease progression in this transgenic model and as a common histopathologic finding in clinical ALS (Figure 5N) (Neumann et al., 2009; Suk and Rousseaux, 2020; Riva et al., 2022). At 3 months of age, pTDP-43 levels in ALS mice treated with vehicle were elevated (2.16 ± 0.23-fold increase) compared with levels in WT mice, as quantified by ELISA. Treatment with ATH-1105 10 mg/kg or 20 mg/kg significantly decreased this pathological protein load in the sciatic nerve of ALS mice (1.49 ± 0.11-fold change from WT for 20 mg/kg). These results, together with findings from plasma biomarkers, point to potent effects of ATH-1105 *in vivo* against three key aspects of ALS disease progression: inflammation, neurodegeneration, and protein pathology.

### ATH-1105 protected against axon degeneration, demyelination, and axonal pTDP-43 accumulation in the sciatic nerve

**Figure 6:**
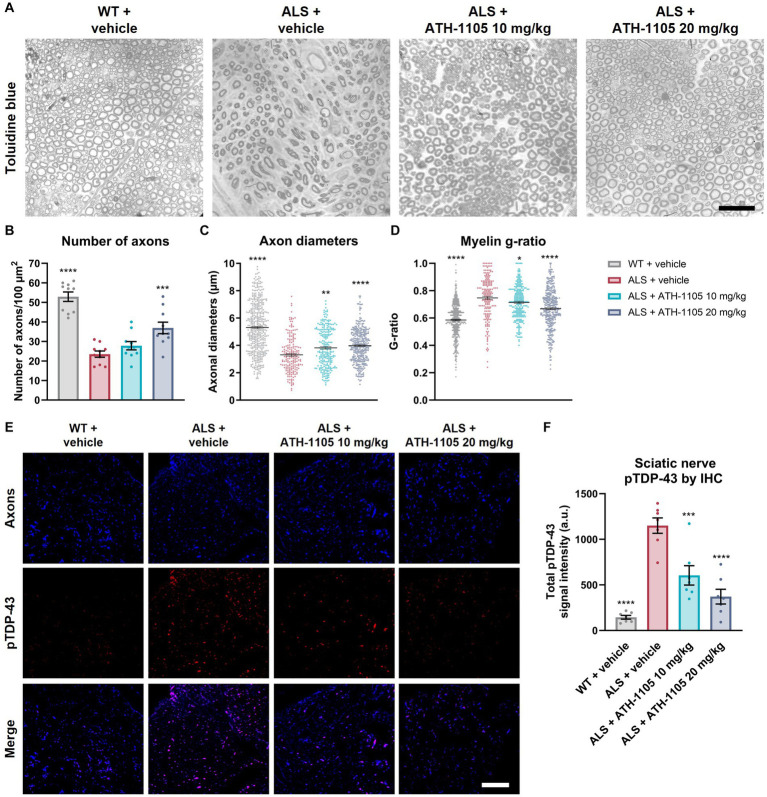
ATH-1105 protects against axon degeneration and demyelination in ALS mice. **(A)** Representative images of sciatic nerve semithin cross sections stained with toluidine blue. Graphical representation of **(B)** number of axons per area, **(C)** distribution of axon diameters, and **(D)** distribution of myelin g-ratio (maximum value, 1.0). Scale bar is 10 μm. **(E)** Representative fluorescence microscopy images of cross sections of sciatic nerve, showing axons, stained for Tuj1 (blue), pTDP-43, stained for phospho-TDP-43 (Ser409/410) (red), and merged images. Scale bar is 50 μm. **(F)** Quantification of total pTDP-43 from immunohistochemical (IHC) analysis. Data are presented as mean ± SEM. Statistical significance was determined via one-way ANOVA with Dunnett’s multiple comparisons versus ALS + vehicle. *p < 0.05, **p < 0.01, ***p < 0.001, ****p < 0.0001; *n* = 10 mice per group for **(B–D)**, *n* = 7 mice per group for **(F)**.

Toluidine blue staining of the sciatic nerve was performed at study termination to determine the impact of 2 months of daily ATH-1105 treatment on nerve structure and integrity in ALS mice (Figures 6A–D). A marked decrease in the average number of axons, axonal diameters, and myelin thickness was observed in the ALS + vehicle group compared with the WT + vehicle group at 3 months of age. ALS mice treated daily with ATH-1105 20 mg/kg for 2 months presented with significantly more axons per area compared with ALS mice treated with vehicle (Figure 6B). The selective loss of large-diameter myelinated axons observed in ALS mice was significantly mitigated by treatment with ATH-1105 10 mg/kg or 20 mg/kg, resulting in higher average axon diameters in these groups (Figure 6C). The g-ratio, calculated as the ratio of the inner diameter of the axon to the diameter of the axon plus the myelin sheath, is used as an index of axonal myelination (Chomiak and Hu, 2009). The theoretical g-ratio for optimal conduction speed in peripheral nerve fibers is approximately 0.6 (Chomiak and Hu, 2009); higher ratios that approach the maximum of 1.0 are indicative of nerve damage and demyelination. The average g-ratio was significantly higher for mice in the ALS + vehicle group at 3 months of age compared with mice in the WT + vehicle group. In ALS mice, daily treatment with ATH-1105 10 mg/kg or 20 mg/kg yielded favorably lower g-ratios than in mice in the vehicle-treated ALS group (Figure 6D). Together, these histopathologic findings point to a dose-dependent protection of axonal integrity and myelination in ALS mice treated with ATH-1105 over the period of 2 months.

To confirm the axonal localization of the protein aggregates initially detected by ELISA, we performed immunohistochemical staining for pTDP-43 in the sciatic nerve (Figure 6E). Co-staining sciatic nerve cross sections with an axonal label (Tuj1 antibody against beta-tubulin III) and a label for aggregated pTDP-43 (anti–pTDP-43 [Ser409/410]) revealed increased levels of pTDP-43 in axons of 3-month-old ALS mice. This extranuclear protein accumulation was significantly and dose-dependently reduced in animals treated for 2 months with ATH-1105 10 mg/kg or 20 mg/kg (Figure 6F).

### ATH-1105 extended survival in ALS mice

**Figure 7:**
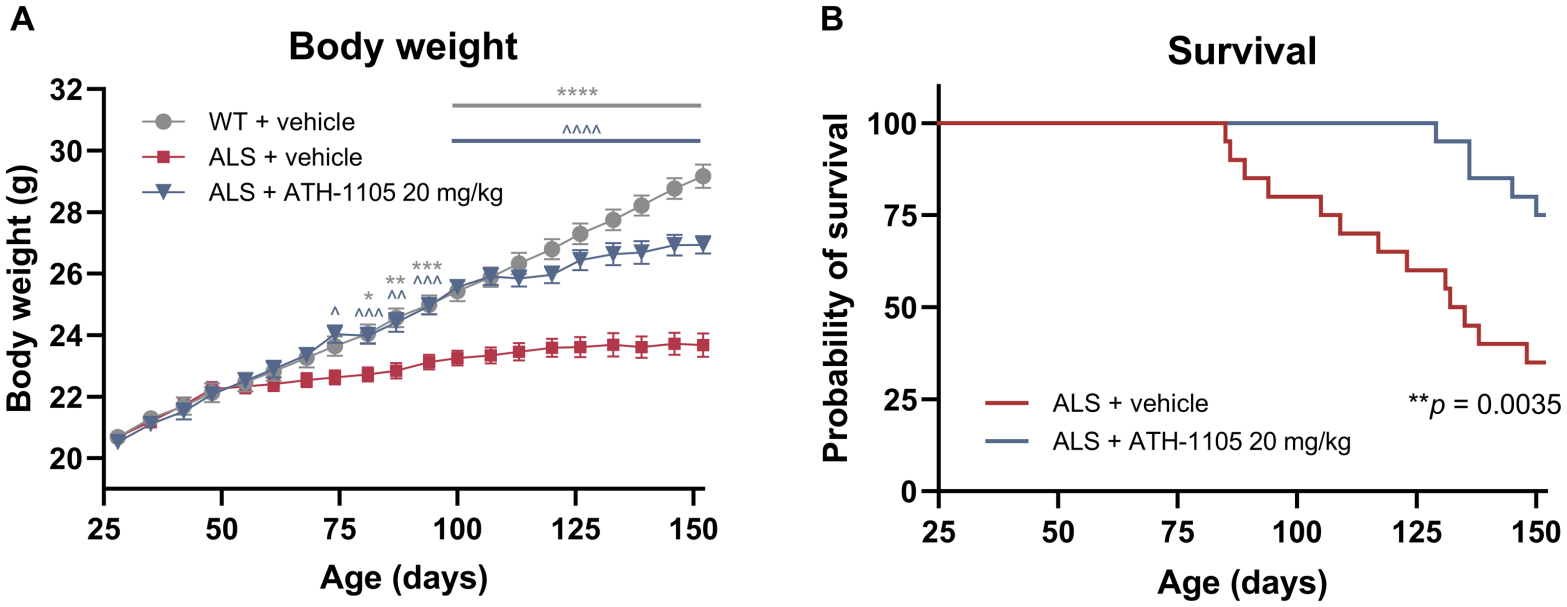
ATH-1105 normalizes body weight and prolongs survival in ALS mice. **(A)** Graphical representation of body weight in grams, collected approximately every 7 days from 1 month of age (28 days old). Data are presented as mean ± SEM. Statistical significance determined by mixed-effects model analysis followed by Dunnett’s multiple comparisons. “*” represents WT + vehicle versus ALS + vehicle comparisons. “^” represents ALS + ATH-1105 20 mg/kg versus ALS + vehicle comparisons. The following applies to all symbols: *p < 0.05, **p < 0.01, ***p < 0.001, ****p < 0.0001. **(B)** Graphical representation of probability of survival by age, in days (maximum of 152 days old). Data are presented as Kaplan–Meier survival probability curves. Statistical significance was determined by log-rank (Mantel-Cox) test; *n* = 20 mice per group at experiment start.

Using a separate cohort of animals, the impact of ATH-1105 treatment on survival in male Prp-TDP43^A315T^ ALS mice was assessed. For the ATH-1105 treatment in this study, we selected the dose of 20 mg/kg as the most efficacious dose previously identified (Figures 5, 6). Animals were given once-daily oral treatment with ATH-1105 or vehicle from 1 month of age (28 days old) until a maximum of 5 months of age (152 days old). WT mice treated with vehicle for the experiment duration were included for body weight comparison purposes but were not included in the survival analyses (no mortality was expected or observed in the WT + vehicle group). Corroborating our previously described findings, the assessment of body weight over time showed that ATH-1105 treatment results in significant and sustained protection of normal body weight levels in ALS mice (Figure 7A). Kaplan–Meier survival analysis over the study duration revealed a significant increase in survival probability over time in ALS mice treated with daily ATH-1105 compared with vehicle-treated ALS mice (Figure 7B). The time to the first mortality event in the ALS mice was delayed from 85 days of age in vehicle-treated animals to 129 days of age in ATH-1105–treated animals. Additionally, the final percentage survival rate at 152 days of age was increased from 35% in the ALS + vehicle group to 75% in the ALS + ATH-1105 20 mg/kg group. In all, data from this study confirm that the neuroprotective benefits observed with ATH-1105 treatment in preclinical models of ALS translate to significantly prolonged survival times in Prp-TDP43^A315T^ mice.

### Delayed intervention with ATH-1105 slowed further ALS-related disease progression from time of treatment onset in ALS mice

**Figure 8:**
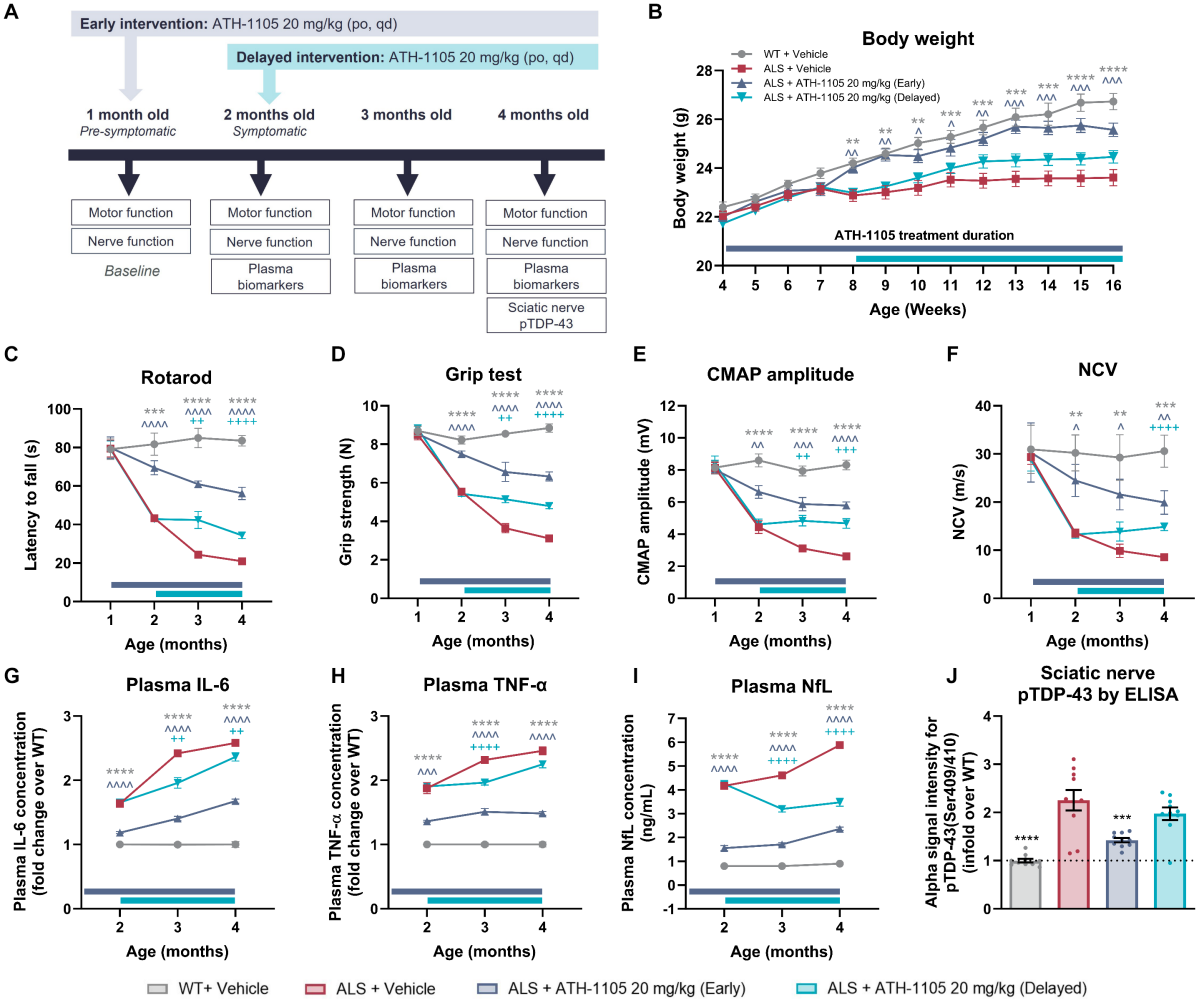
Delayed intervention with ATH-1105 reduces further disease progression in ALS mice. **(A)** Experiment timeline. **(B)** Graphical representation of body weight in grams measured every 3 days from 1 month of age (4 weeks old). Graphical representation of motor function as indicated by **(C)** rotarod latency to fall and **(D)** grip strength. Graphical representation of nerve function as indicated by **(E)** CMAP amplitude and **(F)** NCV. Graphical representation of plasma levels of **(G)** IL-6, **(H)** TNF-α, and **(I)** NfL. Horizontal bars above *x*-axis in **(B–I)** represent ATH-1105 20 mg/kg treatment duration for early intervention (dark blue, top; 1–4 months of age) and delayed intervention (teal, bottom; 2–4 months of age). **(J)** Graphical representation of pTDP-43 levels. Data are presented as mean ± SEM. Statistical significance was determined via mixed-effects model with Dunnett’s multiple comparisons. “*” represents WT + vehicle versus ALS + vehicle comparisons. “^” represents ALS + ATH-1105 20 mg/kg (early) versus ALS + vehicle comparisons. “+” represents ALS + ATH-1105 20 mg/kg (delayed) versus ALS + vehicle comparisons. The following applies to all symbols: *p < 0.05, **p < 0.01, ***p < 0.001, ****p < 0.0001; n = 10 mice per group.

In a final, separate study, we assessed whether any of the previously described preclinical benefits of ATH-1105 would be preserved if treatment initiation was delayed. Male Prp-TDP43^A315T^ transgenic mice were asymptomatic at 1 month of age and exhibited a steep decline in neuromotor function by the age of 2 months (as previously shown in Figure 5). Therefore, we administered daily oral vehicle or ATH-1105 (20 mg/kg) in mice beginning at 1 month of age (early intervention ATH-1105) or 2 months of age (delayed intervention ATH-1105) until a final time point of 4 months of age (Figure 8A). The early intervention ATH-1105 group performed similarly to the dose optimization study presented herein across all metrics (body weight, motor function, nerve function, and plasma and sciatic nerve biomarkers). Although a trend can be observed in the direction of body weight change after treatment onset, delayed intervention with ATH-1105 did not result in statistically significant improvement in the body weight of ALS mice (Figure 8B). However, in behavioral tests, a significant slowing of further deterioration in motor function was observed in the rotarod (Figure 8C), grip test (Figure 8D), Kondziela screen test (Supplementary Figure S3A), and balance beam test (Supplementary Figure S3B) following delayed intervention with ATH-1105. Similarly, electrophysiological measurements of nerve function showed significant protection against further decreases in CMAP amplitude (Figure 8E) and NCV (Figure 8F) from the time of ATH-1105 treatment onset. This suggests that ATH-1105 has preclinical efficacy in slowing ALS-related neuromuscular deterioration, even when treatment initiation is delayed to a more advanced point in disease progression.

To gain insight into the temporal dynamics of biomarkers as a function of ATH-1105 treatment initiation, plasma biomarkers were assessed at multiple time points (2, 3, and 4 months of age) in this study (Figures 8G–I). We found that levels of markers of inflammation (IL-6, Figure 8G; TNF-α, Figure 8H) and neurodegeneration (NfL, Figure 8I) were already elevated in ALS mice treated with vehicle by the first assessed time point of 2 months of age. In ALS mice that underwent delayed intervention with ATH-1105 (treatment starting at 2 months of age), the level of inflammatory biomarkers at the next evaluated time points was reduced relative to those in vehicle-treated ALS mice, potentially indicating a slowing in the progression of the inflammatory phenotype (Figures 8G,H). Notably, a robust decrease in plasma NfL levels was observed in response to delayed intervention with ATH-1105, which remained attenuated for the rest of the study, despite the continued increase in NfL concentrations in the ALS + vehicle group (Figure 8I). These findings suggest potent neuroprotective effects with ATH-1105 treatment even when administered at a delayed timepoint corresponding to a later stage in ALS disease progression. Interestingly, the beneficial effects of delayed intervention ATH-1105 occurred in the absence of a significant reduction in sciatic nerve pTDP-43 levels, suggesting that treatment with ATH-1105 can slow ALS-related neurodegeneration even in the context of high levels of pathological protein accumulation (Figure 8J).

## Discussion

The current work demonstrates that ATH-1105, a small-molecule positive modulator of the neurotrophic HGF system, has potent neuroprotective and anti-inflammatory effects in preclinical models, demonstrating benefit across several key mechanisms of ALS-related pathogenesis. By enhancing HGF-mediated phosphorylation of MET, ATH-1105 and other positive modulators of HGF activate pro-survival signaling cascades that counteract pathological hallmarks of neurodegeneration (Johnston et al., 2023). Our data show that ATH-1105 protects various cell culture systems from glutamate-mediated toxicity and its pathological alterations, which include mitochondrial dysfunction, apoptotic signaling, TDP-43 mis-localization, astrocyte dysfunction, NMJ abnormalities, neurite degeneration, and motor neuron death. *In vivo*, ATH-1105 treatment preserves motor and nerve function, reduces markers of inflammation and neurodegeneration when given as an early or delayed intervention, and significantly extends survival in an ALS mouse model. Together, our data demonstrate the potential of ATH-1105 to address the broad scope of pathological mechanisms associated with neurodegenerative diseases, including ALS.

Although various pathological mechanisms are associated with ALS, substantial attention has focused on glutamate excitotoxicity as a central component of ALS pathology (Corona et al., 2007; Foran and Trotti, 2009; Blasco et al., 2014). Glutamate excitotoxicity occurs when excess glutamate in the extracellular space leads to abnormal activation of glutamate receptors, resulting in elevated levels of intracellular calcium ions (Ca^2+^), upregulation of reactive oxygen species, mitochondrial dysfunction, apoptotic signaling, protein aggregation, and eventual cell death (Corona et al., 2007; Boussicault et al., 2020; Obrador et al., 2020). ATH-1105 treatment protected motor neurons and preserved neurite networks across three different cell systems exposed to glutamate excitotoxicity (spinal motor neuron, motor neuron–astrocyte, and motor neuron–muscle cultures), thereby showing the ability to address a central component of ALS pathology in preclinical models. Mechanistic insight regarding motor neuron protection may be deduced from our observations of intracellular events. Specifically, treatment with ATH-1105 rescued mitochondrial function and reduced apoptotic signaling (assessed via activated caspase-3) in spinal motor neurons after glutamate injury. Such effects may be driven by the ability of ATH-1105 to exert anti-apoptotic and pro-survival effects via activation of AKT and ERK signaling. This line of reasoning is consistent with literature that reports on the anti-apoptotic effects of HGF via AKT and ERK (Xiao et al., 2001).

In addition to motor neuron protection, ATH-1105 also elicited beneficial effects on non-neuronal cells (i.e., astrocytes and microglia), an important observation given the contribution of non–cell-autonomous processes to the pathogenesis of ALS (Van Harten et al., 2021). In neuron-astrocyte cocultures, ATH-1105 significantly reduced astrocyte reactivity, a major contributor to motor neuron injury and degeneration (Yamanaka and Komine, 2018; Vaz et al., 2021). Furthermore, treatment with ATH-1105 increased the expression of the glutamate transporter EAAT2, which is a key player in the maintenance of physiological glutamate levels and is reduced in people with ALS (Rothstein et al., 1995; Rosenblum and Trotti, 2017). Neuroinflammation also plays a major role in catalyzing overall ALS disease progression. Microglia are the resident immune cells of the CNS, and their pathological activation releases a plethora of inflammatory mediators that damage motor neurons. For instance, increasing evidence implicates microglial NLRP3 inflammasome activation and subsequent release of IL-1β in ALS pathogenesis (Deora et al., 2020; Holbrook et al., 2021; Moreno-García et al., 2021). In this regard, the ability of ATH-1105 to induce anti-inflammatory effects in cultured immune cells, such as reduction of NLRP3 expression and IL-1β secretion, provides another mechanism by which ATH-1105 may mitigate ALS pathology.

Another consistent hallmark of ALS is the breakdown and dysfunction of the NMJ, which results in the progressive loss of functional neuromuscular crosstalk, eventually leading to muscle atrophy and paralysis (Cappello and Francolini, 2017; Lépine et al., 2022; Verma et al., 2022). Therefore, interventions that can preserve NMJ integrity via promotion of motor neuron health and/or maintenance of acetylcholine receptor signaling may be well suited to use in addressing the NMJ dysfunction associated with ALS. In an *in vitro* NMJ model, we show that ATH-1105 treatment increased the clustering of acetylcholine receptors and the number of motor units in a motor neuron–muscle coculture system exposed to excessive glutamate. Such effects, if maintained *in vivo*, may reasonably be expected to translate to improved muscle function, especially in conjunction with the observed neuroprotective and anti-inflammatory actions of ATH-1105.

We selected the Prp-TDP43^A315T^ mouse model of ALS to explore the *in vivo* efficacy of ATH-1105. Although rare in clinical populations, mutations in the *TDP-43* gene (including the *A315T* mutation) and their associated cellular and functional outcomes have been extensively described in the context of ALS and frontotemporal dementia (FTD) (Buratti, 2015). The presence of TDP-43 inclusions within degenerating neurons in most people with ALS speaks to a critical role of this protein in ALS pathogenesis (Scotter et al., 2015; Jeon et al., 2019), even when its dysfunction is not necessarily causative or related to a congenital mutation. In the Prp-TDP43^A315T^ mouse model, we confirmed the presence of ALS-like progressive deterioration in motor function and nerve conduction, as well as in sciatic nerve axonal loss and demyelination. Daily oral treatment with ATH-1105 in this model of ALS resulted in statistically significant and dose-dependent improvement across a battery of assessments for motor performance and nerve structure and function, ultimately resulting in prolonged survival. Importantly, the treatment effects of ATH-1105 in this model occurred congruently across the wide range of disease aspects measured. For instance, the g-ratio for myelin is inversely correlated with NCV (Cercignani et al., 2017), and treatment with ATH-1105 favorably lowered the g-ratio in ALS mice while significantly increasing average NCV. Similarly, total motor axon numbers correlate with CMAP measurements: fewer axons lead to lower CMAP amplitudes (Whittaker, 2012; Han et al., 2022), and treatment with ATH-1105 led to a simultaneous increase in axon numbers and CMAP amplitudes. Although we did not investigate the integrity of NMJ structure and function *in vivo*, CMAP amplitude is strongly influenced by this factor, and our *in vitro* results in motor neuron–muscle cocultures suggest that ATH-1105 may also have a positive impact on this aspect of neuromuscular signaling. Together, the CMAP and NCV findings demonstrate concurrent structural and functional improvement in nerve integrity by ATH-1105 treatment in a mouse model with pathological and functional features relevant to clinical ALS, coinciding with improvement in motor function and overall survival.

The beneficial effects of ATH-1105 in the Prp-TDP43^A315T^ mouse model of ALS were further highlighted by dose-dependent reductions of the levels of inflammatory protein markers related to disease progression. Elevated levels of IL-6 in the serum and CSF have been reported in people with ALS, positively correlating with the rate of disease progression (Sekizawa et al., 1998; Ono et al., 2001; Moreau et al., 2005; Mizwicki et al., 2012; Wosiski-Kuhn et al., 2019). A similar increase in levels of TNF-α has been described in plasma, serum, CSF, and CNS tissue of people with ALS; evidence also links this increase to disease progression (Babu et al., 2008; Fukazawa et al., 2013; Brambilla et al., 2016; Brohawn et al., 2016; Guidotti et al., 2021). Treatment with ATH-1105 significantly protected against the pathological increase in both of these translationally relevant inflammatory biomarkers. This suggests that the *in vivo* benefits of ATH-1105 may be driven in part by the anti-inflammatory activity of the compound, in alignment with the properties observed *in vitro* in BV2 microglia, THP-1–differentiated macrophages, and astrocytes. However, additional work is required to confirm effects on these specific cell types *in vivo* in tissues relevant to ALS.

Among the various candidate biomarkers for ALS, NfL is one of the most promising (Lu et al., 2015; Poesen and Van Damme, 2018; Sugimoto et al., 2020). An elevated concentration of NfL in CSF or plasma correlates with disease severity and progression, including in pre-diagnostic ALS (Tortelli et al., 2012; Lu et al., 2015; Gaiani et al., 2017; Gagliardi et al., 2019; Verde et al., 2019; Sugimoto et al., 2020; Bjornevik et al., 2021). We observed a dramatic attenuation of plasma NfL levels with treatment, speaking to the neuroprotective effects of ATH-1105, even within the context of an aggressive genetically driven model of ALS. We further found that ATH-1105 intervention at a delayed time point in disease progression in Prp-TDP43^A315T^ mice partially reversed the elevations in plasma NfL levels from the time of treatment initiation. NfL is an attractive biomarker for use in clinical trials of neurodegenerative diseases, including ALS, as some researchers suggest treatments not protective against increases in NfL lack the potential to modify disease (Kiernan et al., 2021). Measurement of plasma NfL has also been used in trials of several other neurodegenerative diseases, including Parkinson’s disease, multiple sclerosis, and Alzheimer’s disease, further speaking to the validity of this protein as a nonspecific biomarker of neurodegeneration (Gagliardi et al., 2019; Sormani et al., 2019; Committee PaCNSDA, 2022).

As another potential ALS disease biomarker, pTDP-43 accumulation is described in motor nerve biopsies acquired from people living with ALS and may precede axonal degeneration and demyelination (Riva et al., 2022). Interestingly, these accumulations occurred in peripheral tissues, which are more readily accessible for clinical investigation than CNS tissues. The relationship between pTDP-43 levels and pharmacodynamic response in clinical populations is not fully clear, largely because of the impractical and invasive nature of this type of repeated sampling, even when taken from peripheral nerves. Newer techniques, including the quantification of pTDP-43 in neuron-derived exosomes in blood, may further facilitate the use of this protein as a biomarker in the future (Chen et al., 2020). Regardless of its current clinical usefulness, the presence of pTDP-43 in nervous system tissue of most people with ALS implies that this protein abnormality is a critical component of ALS pathogenesis. Reducing accumulation of this protein, including in the periphery, may represent a promising target for novel therapeutics. Therefore, it is compelling that early intervention with ATH-1105 significantly reduced pTDP-43 in the sciatic nerves of Prp-TDP43^A315T^ mice, especially considering that the expression of the mutant protein is genetically driven in this model. Because a direct effect of ATH-1105 on TDP-43 protein expression is not expected as part of the mechanism of action, we hypothesize that the impact of ATH-1105 lies in its ability to interrupt the positive-feedback loop which typically surrounds pathological protein accumulation in neurodegenerative contexts. The presence of misfolded, hyperphosphorylated, or otherwise abnormally processed proteins can lead to increased cellular stress, causing mitochondrial dysfunction and activation of apoptotic pathways, which in turn leads to further dysregulation of protein-processing machinery and exacerbated protein accumulation (Gregersen and Bross, 2010). In primary spinal motor neurons, we observed that ATH-1105 can mitigate mitochondrial dysfunction and caspase-3 activation downstream of glutamate-induced excitotoxicity. We also found almost complete prevention of extranuclear TDP-43 accumulation downstream of a glutamate challenge. These data indicate that the actions of ATH-1105 toward reducing pTDP-43 may be through attenuation of the ongoing cycle of cellular stress and excitotoxicity, which augments protein misprocessing. Interestingly, delayed intervention with ATH-1105 *in vivo* in the Prp-TDP43^A315T^ model significantly slowed further deterioration in motor and nerve function and reduced neurodegeneration (as measured via NfL) from the time of treatment initiation, even in the absence of measurable effects on pTDP-43 levels. This result supports the idea that the therapeutic impact of ATH-1105 in Prp-TDP43^A315T^ mice is largely driven by broad neuroprotective effects which are not solely based on the inhibition of protein accumulation. Overall, these findings suggest that the potential benefits of ATH-1105 against ALS pathogenesis would not be specific to cases primarily driven by *TDP-43* mutations but may be etiologically agnostic.

Future work could further elucidate the mechanisms behind the effects of ATH-1105 in preclinical models of ALS. For example, additional *in vivo* studies are necessary to assess nervous system tissues beyond the sciatic nerve, including brain and spinal cord, to gain a specific understanding of the treatment effects of ATH-1105 on upper and lower motor neurons, as well as on NMJs and the muscles themselves (Kiernan et al., 2011; Cappello and Francolini, 2017). The effects of ATH-1105 on myelinating Schwann cells should also be investigated to gain insight on how treatment may be preserving myelination and whether this is through direct activity in Schwann cells and/or oligodendrocytes. Although results of *in vitro* studies suggest that positive modulation of the HGF system leads to cellular activity in multiple neuron types, astrocytes, microglia, and macrophage-like cells, further work is necessary to confirm target engagement in neurons and disease-relevant assays. Similarly, evidence of *in vivo* target engagement, such as tissue levels of pMET, pERK, and/or pAKT after ATH-1105 treatment, should be sought, although the transient temporal nature of HGF/MET system activation renders this endeavor technically challenging. Though the translatability of preclinical models of ALS has been an obstacle in drug development, our findings across multiple preclinical models are encouraging, indicating that the observed effects may not be model- or mutation-specific. It should be noted, however, that the Prp-TDP43^A315T^ mouse model of ALS may be limited by the absence of substantial lower motor neuron loss and lack of TDP-43 nuclear depletion, both of which can be critical aspects of clinical ALS. Interestingly, this model does feature extranuclear pTDP-43 accumulation in peripheral as well as central axons, and may therefore be a useful tool for evaluating this important disease component. Future studies should be aimed at confirming the broad applicability of ATH-1105 to ALS derived from varying causes, including investigating efficacy in animal models, primary cultures, and human induced pluripotent stem cells (iPSCs) harboring ALS-related mutations different than the TDP-43^A315T^ mutation studied here.

Overall, the studies presented highlight several lines of preclinical evidence that support the potential beneficial effects of ATH-1105 in ALS. The evidence brought forth by this work via multiple distinct assays, which span *in vitro* and *in vivo* systems, shows positive, dose-dependent, statistically significant effects of the HGF positive modulator ATH-1105 in ALS-relevant disease models. Given these data and the critical unmet medical need and devastating disease progression of ALS, we are encouraged to continue the development of ATH-1105 as a potential therapeutic agent for people living with ALS.

## Data availability statement

The original contributions presented in the study are included in the article/Supplementary material, further inquiries can be directed to the corresponding author.

## Ethics statement

Ethical approval was not required for the studies on humans in accordance with the local legislation and institutional requirements because only commercially available established cell lines were used. The animal study was approved by the Institutional Animal Ethics Committee of Sai Life Sciences Limited, the Institutional Animal Ethics Committee of Aurigene Pharmaceutical Services Limited, and the Animal Studies Committee of Languedoc Roussillon. The study was conducted in accordance with the local legislation and institutional requirements.

## Author contributions

A-AB: Conceptualization, Data curation, Formal analysis, Project administration, Visualization, Writing – original draft, Writing – review & editing. SR: Conceptualization, Data curation, Formal analysis, Project administration, Visualization, Writing – original draft, Writing – review & editing. KK: Conceptualization, Data curation, Formal analysis, Project administration, Writing – review & editing. SS: Conceptualization, Project administration, Writing – review & editing. WW: Conceptualization, Data curation, Formal analysis, Project administration, Visualization, Writing – review & editing. JJ: Conceptualization, Project administration, Supervision, Writing – review & editing. RT: Conceptualization, Supervision, Writing – review & editing. LS: Conceptualization, Writing – review & editing. HM: Writing – review & editing. KC: Conceptualization, Supervision, Writing – review & editing.
