# Supplementary material for: ATH-1105, a small-molecule positive modulator of the neurotrophic HGF system, is neuroprotective, preserves neuromotor function, and extends survival in preclinical models of ALS
Source: Front Neurosci. 2024 Feb 8;18:1348157. doi: 10.3389/fnins.2024.1348157 (PMC10881713; doi:10.3389/fnins.2024.1348157)
Supplement: Supplementary file 1 [file Table_1.DOCX]

# Additional file 1

**Supplementary Table S1.** Key resources table

| **Reagent or resource** | **Source** | | **Identifier** | |
| --- | --- | --- | --- | --- |
| **Antibodies** |  | |  | |
| Advanced phospho-ERK Eu cryptate antibody | Cisbio | | 64AERPET | |
| Advanced phospho-ERK D2 antibody | Cisbio | | 64AERPET | |
| Advanced phospho-AKT Eu cryptate antibody | Cisbio | | 64AKSPET | |
| Advanced phospho-AKT D2 antibody | Cisbio | | 64AKSPET | |
| Mouse monoclonal anti–MAP-2 antibody | Sigma-Aldrich | | M4403-.2ML | |
| Alexa Fluor 488 goat anti-mouse IgG | Sigma-Aldrich | | SAB4600042-250ul | |
| Rabbit polyclonal anti-nuclear TDP-43 antibody | Ozyme | | 3448s | |
| Alexa Fluor 568 goat anti-rabbit antibody | Sigma-Aldrich | | SAB4600084-250ul | |
| **Chemicals, peptides, and recombinant proteins** | | | | |
| Hoechst solution | Sigma-Aldrich | | 94403 | |
| MitoTracker Red CMXRos | Cell Signaling Technology | | 9082S | |
| HGF | R&D Systems | | 2207-HG | |
| Phosphatase arrest | G-Biosciences | | 786-647 | |
| **Critical commercial assays** | | | | |
| PathScan phospho-Met (Tyr1234/1235) Sandwich ELISA kit | Cell Signaling Technology | | 7227C | |
| Advanced phospho-ERK (Thr202/Tyr204) cellular kit | Cisbio | | 64AERPET | |
| Phospho-AKT (Ser473) cellular kit | Cisbio | | 64AKSPET | |
| CellTiter-Glo assay | Promega Corporation | | G7570 | |
| Mouse IL-6 ELISA kit | Sigma-Aldrich | | RAB0308 | |
| Mouse TNF-α ELISA kit | Sigma-Aldrich | | RAB0477 | |
| Mouse NF-L ELISA kit | Novus Biologicals | | NBP2-80299 | |
| **Experimental models: cell lines** | | | | |
| HEK293 | ATCC | | CRL-1573 | |
| **Experimental models: organisms** | | | | |
| TDP-43^A315T^ transgenic mice | Jackson Laboratory | | #010700 | |
| Sprague Dawley rats | Vivo Bio Tech Limited | | – | |
| Sprague Dawley rats | Janvier Labs | | – | |
| **Software** | |  | |  |
| ImageJ | NIH | | https://imagej.nih.gov/ij/ | |
| Envision workstation (version 1.14.3049.528) | PerkinElmer | | https://www.perkinelmer.com/ | |
| GraphPad Prism (version 9.1.2, 10.0.1) | GraphPad | | https://www.graphpad.com/ | |
| ImageXpress | Molecular Devices | | https://www.moleculardevices.com/products/cellular-imaging-systems | |

# Supplementary Figures


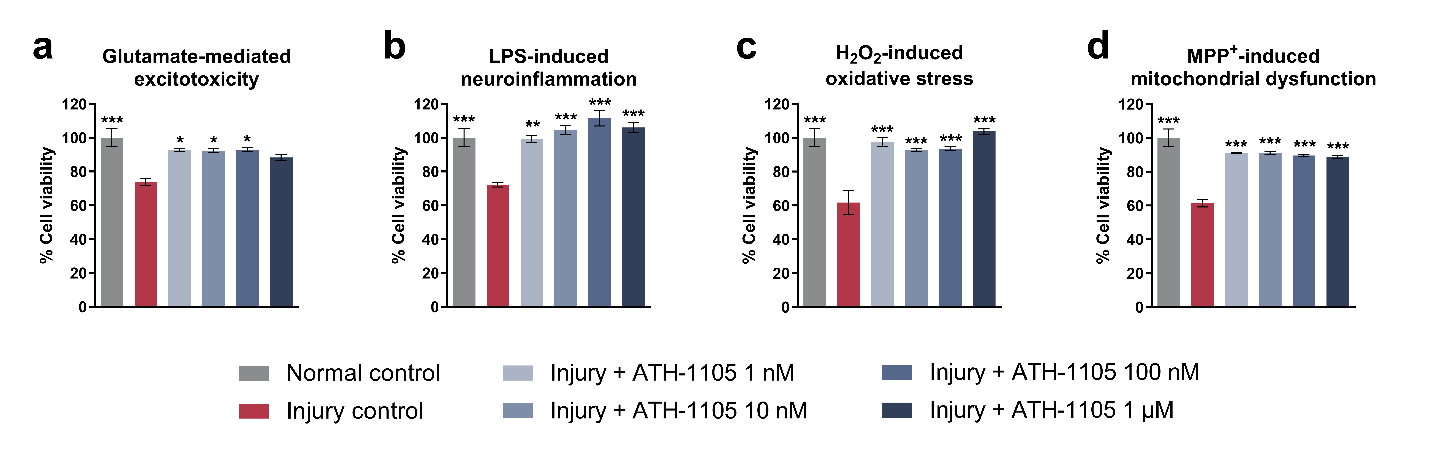


**Supplementary Fig. S1 ATH-1105 is neuroprotective in primary culture of cortical neurons.** Primary cortical neurons treated with ATH-1105 (in addition to each respective neurotoxin) showed significant increase in cell survival (via CellTiter-Glo; Promega Corporation) against neurotoxic insults, including **a** glutamate-induced excitotoxicity, **b** LPS-induced inflammation, **c** H_2_O_2_-induced oxidative stress, and **d** MPP^+^-induced mitochondrial dysfunction. Data are presented as mean ± SEM; n = 4 (1 culture). Statistical significance was determined by one-way ANOVA with Tukey’s test. **P* < 0.05, ***P* < 0.01, ****P* < 0.001 versus respective injury.


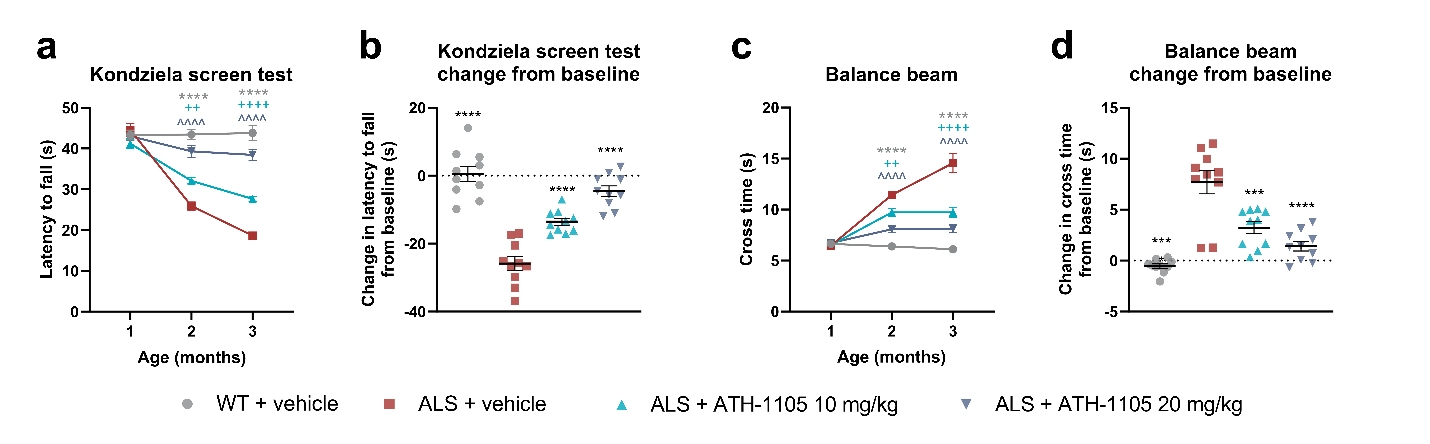


**Supplementary Fig. S2 Additional neuromotor assessments in ALS mice treated with ATH-1105 at 10 or 20 mg/kg.** Graphical representation of motor function as measured by **a** Kondziela screen test over time and as **b** change from baseline in latency to fall and as measured by **c** balance beam over time and as **d** change from baseline in cross time. Data are presented as mean ± SEM. Statistical significance was determined via **a,c** two-way ANOVA or **b,d** one-way ANOVA with Dunnett’s multiple comparison test versus ALS + vehicle. “*” represents WT + vehicle versus ALS + vehicle comparisons, “+” represents ALS + ATH-1105 10 mg/kg versus ALS + vehicle comparisons, and “^” represents ALS + ATH-1105 20 mg/kg versus ALS + vehicle. The following applies to all symbols: **P* < 0.05, ***P* < 0.01, ****P* < 0.001, *****P* < 0.0001; n = 10 mice per group.


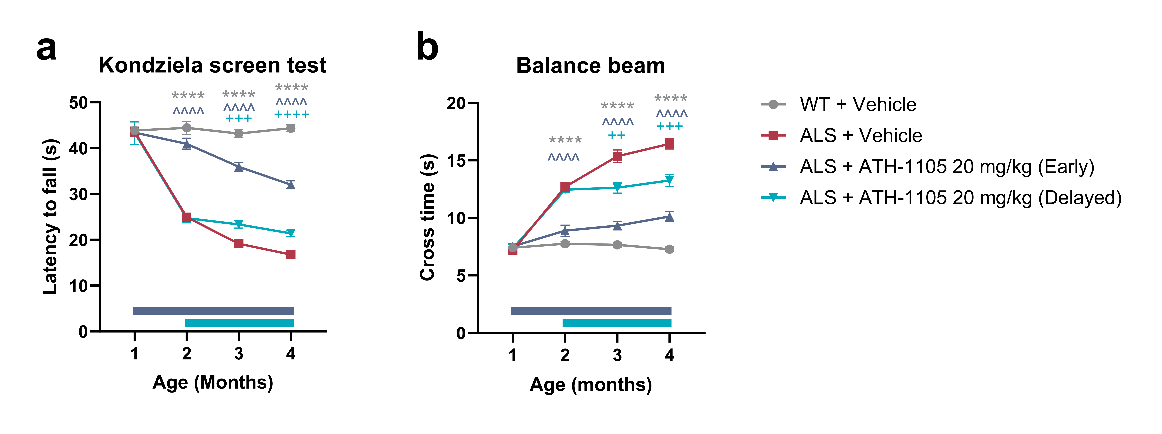


**Supplementary Fig. S3 Additional neuromotor assessments in ALS mice after early or delayed intervention with ATH-1105.** Graphical representation of **a** latency to fall in Kondziela screen test and **b** cross time in balance beam from 1 to 3 months of age. Horizontal bars above *x*-axis represent ATH-1105 treatment duration for early intervention (dark blue, top; 1–4 months of age) and delayed intervention (teal, bottom; 2–4 months of age). Data are presented as mean ± SEM. Statistical significance was determined via **a,b** two-way ANOVA with Dunnett’s multiple comparison test versus ALS + vehicle. “*” represents WT + vehicle versus ALS + vehicle comparisons, “^” represents ALS + ATH-1105 20 mg/kg (early) versus ALS + vehicle comparisons, and “+” represents ALS + ATH-1105 20 mg/kg (delayed) versus ALS + vehicle comparisons. The following applies to all symbols: **P* < 0.05, ***P* < 0.01, ****P* < 0.001, *****P* < 0.0001; n = 10 mice per group.
